# Threshold-free genomic cluster detection to track transmission pathways in health-care settings: a genomic epidemiology analysis

**DOI:** 10.1016/S2666-5247(22)00115-X

**Published:** 2022-07-05

**Authors:** Shawn E Hawken, Rachel D Yelin, Karen Lolans, Ali Pirani, Robert A Weinstein, Michael Y Lin, Mary K Hayden, Evan S Snitkin

**Affiliations:** Department of Microbiology and Immunology, University of Michigan Medical School, Ann Arbor, MI, USA; Department of Internal Medicine, Division of Infectious Diseases, Rush University Medical Center, Chicago, IL, USA; Department of Internal Medicine, Division of Infectious Diseases, Rush University Medical Center, Chicago, IL, USA; Department of Microbiology and Immunology, University of Michigan Medical School, Ann Arbor, MI, USA; Department of Internal Medicine, Division of Infectious Diseases, Rush University Medical Center, Chicago, IL, USA; Department of Internal Medicine, Division of Infectious Diseases, Rush University Medical Center, Chicago, IL, USA; Department of Internal Medicine, Division of Infectious Diseases, Rush University Medical Center, Chicago, IL, USA; Department of Microbiology and Immunology, Internal Medicine Division of Infectious Diseases, University of Michigan Medical School, Ann Arbor, MI, USA

## Abstract

**Background:**

A crucial barrier to the routine application of whole-genome sequencing (WGS) for infection prevention is the insufficient criteria for determining whether a genomic linkage is consistent with transmission within the facility. We evaluated the use of single-nucleotide variant (SNV) thresholds, as well as a novel threshold-free approach, for inferring transmission linkages in a high-transmission setting.

**Methods:**

We did a retrospective genomic epidemiology analysis of samples previously collected in the context of an intervention study at a long-term acute care hospital in the USA. We performed WGS on 435 isolates of *Klebsiella pneumoniae* harbouring the *bla_KPC_* carbapenemase (KPC-*Kp*) collected from 256 patients through admission and surveillance culturing (once every 2 weeks) of almost every patient who was admitted to hospital over a 1-year period.

**Findings:**

Our analysis showed that the standard approach of using an SNV threshold to define transmission would lead to false-positive and false-negative inferences. False-positive inferences were driven by the frequent importation of closely related strains, which were presumably linked via transmission at connected health-care facilities. False-negative inferences stemmed from the diversity of colonising populations that were spread among patients, with multiple examples of hypermutator strain emergence within patients and, as a result, putative transmission links separated by large genetic distances. Motivated by limitations of an SNV threshold, we implemented a novel threshold-free transmission cluster inference approach, in which each of the acquired KPC-*Kp* isolates were linked back to the imported KPC-*Kp* isolate with which it shared the most variants. This approach yielded clusters that varied in levels of genetic diversity but where 105 (81%) of 129 unique strain acquisition events were associated with epidemiological links in the hospital. Of 100 patients who acquired KPC-*Kp* isolates that were included in a cluster, 47 could be linked to a single patient who was positive for KPC-*Kp* at admission, compared with 31 and 25 using 10 SNV and 20 SNV thresholds, respectively. Holistic examination of clusters highlighted extensive variation in the magnitude of onward transmission stemming from more than 100 importation events and revealed patterns in cluster propagation that could inform improvements to infection prevention strategies.

**Interpretation:**

Our results show how the integration of culture surveillance data into genomic analyses can overcome limitations of cluster detection based on SNV-thresholds and improve the ability to track pathways of pathogen transmission in health-care settings.

**Funding:**

US Center for Disease Control and Prevention and University of Michigan.

## Introduction

Health-care-associated infections are a major threat to patient safety.^1^ Despite increased attention to infection prevention in health-care settings, cross-transmission between patients admitted to hospital still occurs, suggesting that pathways of nosocomial transmission remain poorly understood.^2^ Integration of genomics with traditional hospital epidemiological investigations has proved powerful in the identification of routes of health-care-associated infection transmission,^3-5^ bringing hope that broad deployment of sequencing can direct improvements in infection prevention practices and drive hospital resources towards effective interventions. However, a major barrier to the use of genomics to track the spread of infections is the absence of clear, robust, and generalisable criteria to assess whether two patients are linked by transmission within a given health-care facility.

The current gold standard for inferring transmission is the imposition of a single-nucleotide variant (SNV) threshold, above which transmission in the facility is deemed improbable, and below which transmission is deemed probable.^6-8^ Optimal SNV thresholds are typically identified on the basis of the quantification of shared health-care exposures among individuals linked at different thresholds.^7-9^ However, there are numerous studies suggesting that single SNV thresholds might lead to inaccuracies in transmission inferences, particularly for tracking the spread of the successful epidemic strains that are responsible for most antibiotic resistance in health-care settings.^9-13^ One source of false-positive transmission inferences via SNV thresholds is patients harbouring closely related strains owing to transmission at a connected health-care facility. Evidence of the confounding effect of transmission at connected health-care facilities being a substantial issue comes from the observation of closely related strains circulating in health-care facilities that share many patients.^10,12,14^ In other words, although a small SNV distance is informative of recent direct or indirect transmission, the frequent movement of strains between facilities limits the ability to use an SNV threshold to determine where putative transmission events occurred. A second source of false positives is the use of species-wide SNV thresholds, with a 2021 study showing strain variation in evolutionary rates and transmission pathways that hinder generalisability.^15^

In addition to false-positive inferences, there is abundant evidence that the use of SNV thresholds can lead to false-negative inferences, in which actual transmission events are excluded because of higher-than-expected SNV distances. A well established source of large SNV distances between true transmission pairs is genetic variation that arises in a patient during prolonged asymptomatic colonisation.^13,16,17^ In particular, with the common practice of sequencing single isolates from a patient’s colonising population, it becomes possible for patients linked by transmission to harbour isolates as genetically distant as any two members of the source patient’s colonising population. Evidence in support of this issue comes from both the observation of large genetic variation in patients’ colonising populations^17-20^ and the fact that the most accurate SNV thresholds for recent transmission are consistent with diversity that accumulates over years.^9,13^ A second source of greater-than-expected genetic diversity among transmission linkages is homologous recombination between distantly related strains, which can amplify genetic distances if steps are not taken to mask recombinant regions.^21^

Although the aforementioned issues with SNV thresholds are well known, it remains unclear how substantial a barrier they represent to accurate transmission inference in health-care settings. To guide infection prevention, easily interpretable criteria are required; however, there are currently insufficient viable options. In this study, we sought to understand how prominent the theoretical limitations to SNV thresholds are and develop an alternative strategy that relies on genetic context instead of genetic distance to identify patients linked by transmission within a facility.

## Methods

### Study design and participants

Detailed information regarding the study design, intervention bundle, and data collection are available elsewhere^22^ and in the appendix (p 1). A 1-year intervention to prevent colonisation and infection with *Klebsiella pneumoniae* harbouring the bla_KPC_ carbapenemase (KPC-*Kp*) was performed from June 18, 2012, to June 30, 2013, in a long-term acute care hospital (LTACH) in Chicago (IL, USA). To monitor the effectiveness of the intervention admission and every-other-week active surveillance, culturing was performed on all patients from the LTACH. All patients in the facility were eligible to participate (ie, there were no exclusion criteria). Bundled intervention comprised screening patients for KPC-*Kp* rectal colonisation on admission and every other week, contact isolation and geographical separation of patients who were postive for KPC-*Kp* in ward cohorts or single rooms, bathing all patients daily with chlorhexidine gluconate, and education and adherence monitoring of health-care workers. Patients were grouped into categories on the basis of the surveillance culture results. Patients who were either positive at the start of the study or within 3 days of LTACH admission were considered potential sources of KPC-*Kp* importation and onward transmission within the LTACH. Patients who were negative for KPC-*Kp* on their first surveillance culture and then positive for KPC-*Kp* after day 3 of admission were assumed to have acquired KPC-*Kp* in the facility, and were treated as patients with high-confidence acquisitions for method comparisons. If a patient’s first surveillance sample was collected more than 3 days after admission and was positive for KPC-*Kp*, the patient was also assumed to have acquired KPC-*Kp* in the facility for the purposes of the transmission cluster detection algorithm. When a patient who was positive at admission acquired an additional KPC-*Kp* strain (as evidenced by multi-locus sequence type inferred from whole-genome sequencing [WGS] data) during their stay, this was termed a secondary acquisition, and such isolates from patients who were positive at admission were eligible to be included as acquisition isolates for transmission cluster detection (appendix p 1–2). This study was reviewed and approved by both the institutional review boards at Rush University Medical Center (Chicago, IL, USA) and the University of Michigan (Ann Arbor, MI, USA). Informed consent was waived.

To circumvent the absence of a discriminatory SNV threshold, we sought to apply an approach that relies on genetic and epidemiological context, instead of genetic distance, to identify patients linked by transmission in the facility. We took advantage of our comprehensive knowledge of which patients imported and acquired KPC-*Kp* and applied an algorithm in which phylogenetic clusters were identified that grouped each acquisition isolate with the most closely related admission or study-start isolate taken from a patient earlier in the study. This approach attempts to track each acquisition isolate back to the patient who imported it into the facility by identifying the importation isolate with which it shares a most recent common ancestor (appendix p 13).

### Procedures

Details on sample processing, whole genome sequencing, quality control, genome annotation and variant calling are provided in the appendix (pp 2–3). Briefly, after filtering and trimming low-quality reads, variants were identified by mapping reads to ST-specific reference genomes. Threshold-free transmission clusters were identified by grouping each acquisition isolate with the admission isolate with which it shared a most recent common ancestor. The ability of threshold-free clusters to uniquely group acquired isolates to importation isolates was compared with the imposition of a range of SNV thresholds by quantifying the number of importation isolates associated with each acquisition isolate for a given threshold. To evaluate the ability of the threshold-free cluster algorithm to group together patients likely linked by transmission, the spatiotemporal overlap among cluster members was compared with permuted clusters. Clusters were permuted to maintain the same size distribution and number of importation and acquisition patients per cluster. Details on implementation can be found in the appendix (pp 3–4).

### Statistical analysis

Two-sample Kolmogorov–Smirnov tests were used to evaluate the null hypothesis that the distribution of SNV distance between acquisition isolates and their closest admission isolate is the same as for those between pairs of admission isolates. Multinomial tests were used to evaluate the null hypothesis that the mutational frequencies in transmission cluster isolates could be observed by chance given the number of mutations and the mutational frequencies across all isolates of the same sequence type collected in the study. A Wilcoxon rank-sum test was used to evaluate the null hypothesis that the median SNV distances among isolates from the same patient is the same for patients who imported KPC-*Kp* isolates and patients who acquired KPC-*Kp* isolates. Permutation tests were used to evaluate the null hypothesis that spatiotemporal and sequential exposures between patients in transmission clusters were random. p values less than 0·05 were considered statistically significant. All statistics were performed using R stats package, version 3.6.1.

### Role of the funding source

The funders of the study had no role in study design, data collection, data analysis, data interpretation, or writing of the report.

## Results

**Figure 1:**
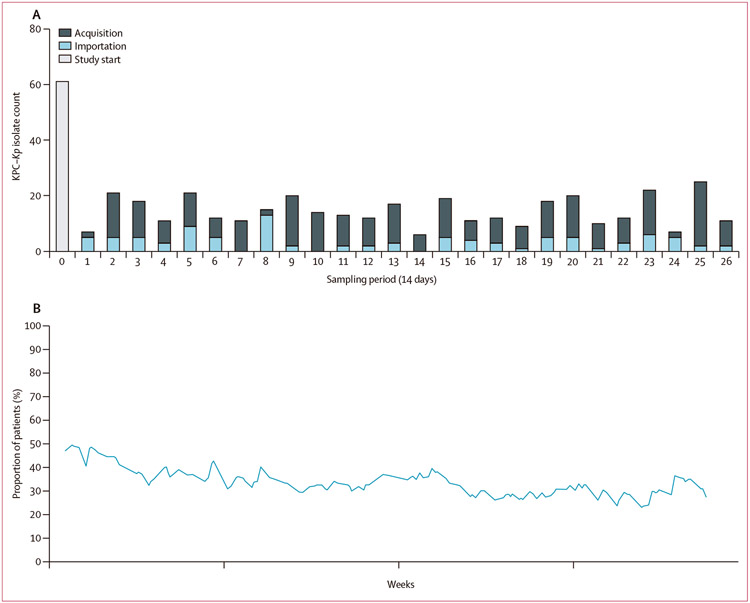
Endemicity of KPC-*Kp* in the LTACH throughout the 1-year study (A) Isolates obtained through rectal surveillance culturing of patients in the LTACH once every 2 weeks. Grey boxes indicate the study-start (0 days) and every two 14-day surveillance periods (28 days) throughout the study. Bars indicate the KPC-*Kp* isolates collected at the beginning of the study (for which importation or acquisition status is not known; light grey, study-start), within 3 days of the patient first entering the facility (blue, importation), or after negative surveillance or more than 3 days after ever being in the LTACH during the study (dark grey, acquisition). (B) KPC-*Kp* prevalence (blue line) is defined as the number of patients presently in the LTACH who are or ever had been surveillance positive for at least one KPC-*Kp* isolate during the study, divided by the number of patients in the facility (daily census) throughout the 1-year study. KPC-*Kp*=*Klebsiella pneumoniae* harbouring the *bla_KPC_* carbapenemase. LTACH=long-term acute care hospital.

**Figure 2:**
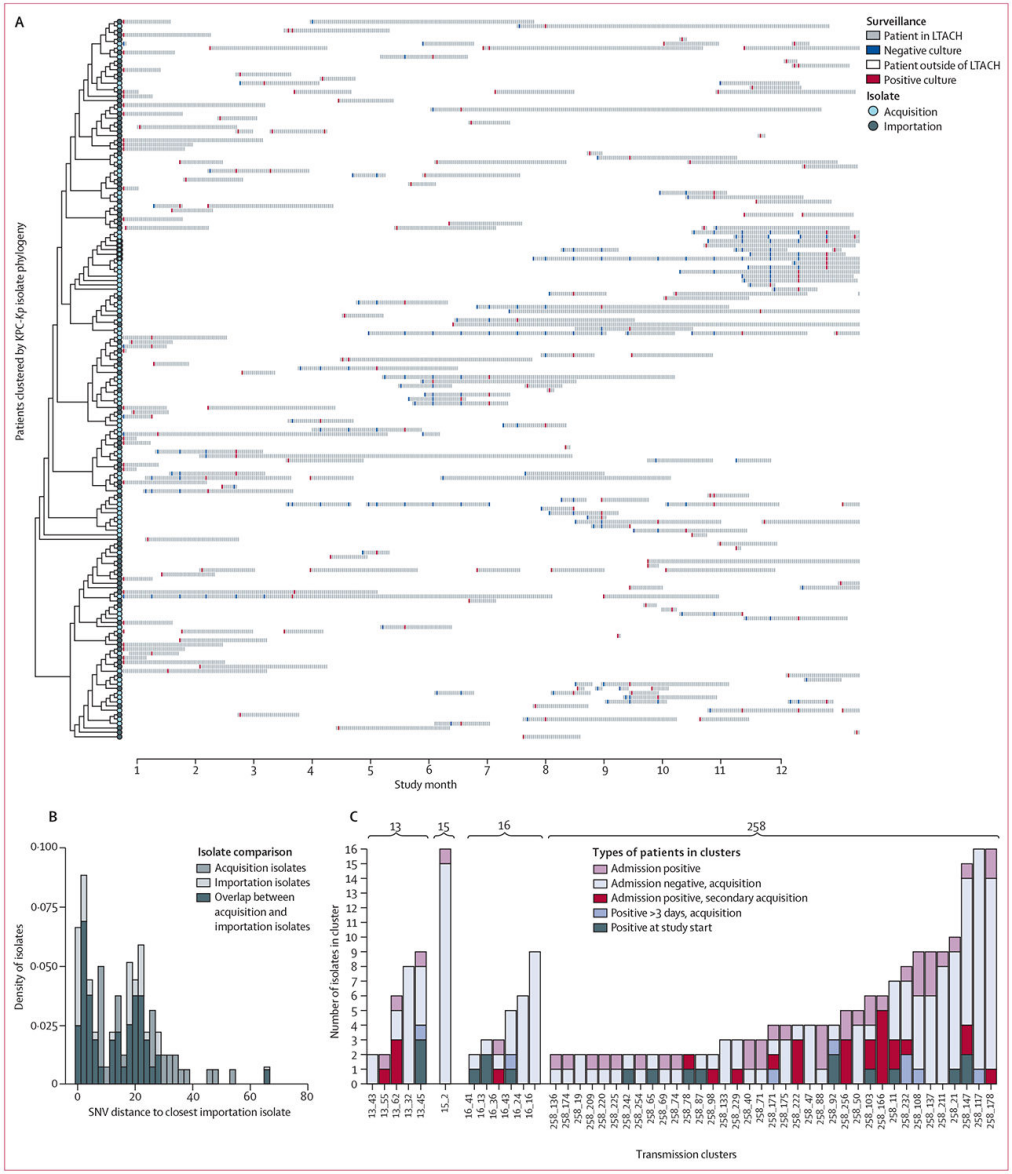
Detection of threshold-free transmission clusters within the LTACH (A) Patient bed trace showing surveillance culture data for patients who tested positive for KPC-*Kp* strain ST258, at any point during the study. A plot illustrating other sequence types detected during the study is shown in the appendix (p 12). (B) Comparison of minimum pairwise SNV distances to the closest imported isolate for acquired and imported isolates. SNV distance versus density (ie, distribution was normalised so total area under curve is the same for acquired and imported distributions) of KPC-*Kp* isolates from ST258 is shown. Light grey bars indicate the minimum distance between isolates collected from patients who acquired KPC-*Kp* colonisation after being in the LTACH for more than 3 days during the admission-positive isolates. Dark grey bars indicate the minimum distance between isolates collected from patients who were positive on admission to the LTACH. The darkest grey colour indicates overlap between the two distributions. Patients who were-positive for KPC-*Kp* on the first day of the study and those who represent a mixture of recent and previous colonisation were considered admission-positive for this analysis so that acquisitions derived from those transmission chains could be linked. The two-sample KS test was used for differences in the distribution of pairwise SNV distances (for ST258, KS statistic=0·16 and p=0·28; for non-ST258, KS statistic=0·21 and p=0·39; for all sequence types combined, KS statistic=0·09 and p=0·81). (C) Distribution of isolates and patients in the 49 transmission clusters detected with the threshold-free approach. Each column represents isolates from one cluster. Admission-positive patients (pink) were patients whose isolate in the cluster was obtained within 3 days of first admission to the facility. Purple indicates isolates obtained from acquisition patients who first acquired KPC-*Kp* colonisation more than 3 days after first admission to the LTACH. Orange indicates isolates from admission-positive patients that were collected more than 3 days after admission to the LTACH, indicating either prolonged colonisation or secondary strain acquisition in the LTACH. Blue indicates patients who were first positive after being in the LTACH for more than 3 days, but from whom no negative swab was collected before first KPC-*Kp* detection. Grey indicates patients who were positive on the first day of the study. The bar across top of figure indicates sequence type of isolates. KPC-*Kp*=*Klebsiella pneumoniae* harbouring the *bla_KPC_* carbapenemase. KS=Kolmogorov-Smirnov. LTACH=long-term acute care hospital. SNV=single nucleotide variant.

On the first day of the 1-year study, all patients (n=83) in the hospital had rectal surveillance cultures to detect colonisation with KPC-*Kp*, which identified 39 colonised patients (46% prevalence). During the rest of the study, admission surveillance detected another 77 patients who were positive within 3 days of first admission and, therefore, presumed to have imported KPC-*Kp* into the facility. Additionally, 128 patients were presumed to have acquired KPC-*Kp* colonisation in the hospital due to having at least one negative surveillance culture before a positive or having been in the facility for more than 3 days before a surveillance culture was taken. Although acquisition and importation fluctuated over time (figure 1A), the overall colonisation prevalence was consistently high, averaging 32% over the course of the year (figure 1B). Of all 256 patients with colonised KPC-*Kp*, classification of the isolates by sequence type revealed that 271 (62%) of the 435 isolates obtained during the study belonged to ST258—the major epidemic lineage of KPC-*Kp* in the USA (appendix p 8), with six other lineages suggesting evidence of intrafacility spread as inferred from their detection in at least one importation and acquisition culture (appendix p 8 and p 11). The seven lineages with putative in-hospital transmission links were imported between one and 83 times each and were the source of two to 104 acquisitions over the course of the study (appendix p 8). Patients harbouring these strains had extensive shared time in the facility (figure 2A, appendix p 12), showing the complexity of deciphering transmission chains in the facility. 242 (96%) of 256 patients, including 139 (92%) of the 151 patients harbouring acquisition isolates, had shared time in the facility with at least one other patient who was positive for a strain matching their acquired sequence type (acquisition isolates) or imported sequence type (importation isolates).

We applied the increased resolution of WGS to discern which patients were linked by cross-transmission in the facility. First, we examined the potential of applying an SNV threshold to identify patients with isolates linked by cross-transmission that occurred in the hospital during the study. One type of error associated with the use of SNV thresholds are false-positive transmission inferences due to the importation of closely related strains that are linked by previous transmission that occurred between patients in a connected health-care facility. To assess the effect of this type of error in our data, we compared the distribution of genetic distances among pairs of isolates imported into the facility with genetic distances among pairs including an imported and acquired isolate. Figure 2B shows that these two distributions are completely overlapping, and that it is commonplace for imported isolates to be related to one another by small genetic distances. Thus, in this high-prevalence endemic setting, there is no SNV threshold that accurately discriminates patients linked by transmission in the facility during the current admission versus during an earlier admission at a connected facility.

To circumvent the absence of an optimal SNV threshold, we used a threshold-free approach that relies on genetic and epidemiological context to group patients acquiring KPC-*Kp* with the most closely related imported isolate. Application of this genomic cluster detection method yielded 49 putative transmission clusters grouping a median of three (range 2–14) patients into clusters representing at least one acquisition event and at least two patients (figure 2C). For 40 (82%) of the 49 clusters, all cluster members could be traced back to a putative importation isolate (table). For seven (78%) of the nine clusters that did not have a patient who was KPC-*Kp*-positive at admission as the first cluster member, spatiotemporal overlap explained all intracluster transmission events, which indicated that only the source of the cluster was not identified (table, appendix p 18). In addition to the 49 transmission clusters, there were 18 (14%) of 128 patients colonised with KPC-*Kp* at the start of the study or on admission surveillance screening whose isolates were not linked with a transmission cluster and, therefore, presumed to not be associated with onward transmission of KPC-*Kp*.

We sought to compare the threshold-free clusters with those identified using different SNV cutoffs. Although we evaluated a range of SNV thresholds, we focus here on 10 SNV and 20 SNVs, given that these have been recommended for KPC-Kp based on recent regional epidemiologic studies.^9,12^ Given that the objective of our study was to identify putative sources of intrafacility transmission, we assessed methods based on how patients who acquired KPC-*Kp* were linked backed to patients importing KPC-*Kp* into the facility (appendix p 15). In addition to finding that patients who acquired KPC-*Kp* were frequently linked back to different admission-positive patients via threshold-free and threshold-based approaches (appendix p 10), there was a trade-off between sensitivity and specificity using SNV thresholds that was mitigated by the threshold-free approach. Examination of the 100 patients with a high-confidence acquisition of KPC-*Kp* (ie, those who did not import a different strain) included in the threshold-free clusters, 36, 47, and 17 were linked to zero, one, and multiple patients who imported KPC-*Kp*, respectively. With a 10-SNV threshold, we found that fewer patients were linked back to putative sources, with 56, 31, and 13 patients who acquired KPC-*Kp* linked back to zero, one, and multiple admission-positive patients, respectively. Conversely, with a 20-SNV threshold, there was a decreased capacity to link acquisitions to a unique admission-positive patient source, with 39, 25, and 36 patients linked back to zero, one, and multiple admission-positive patients, respectively.

**Figure 3:**
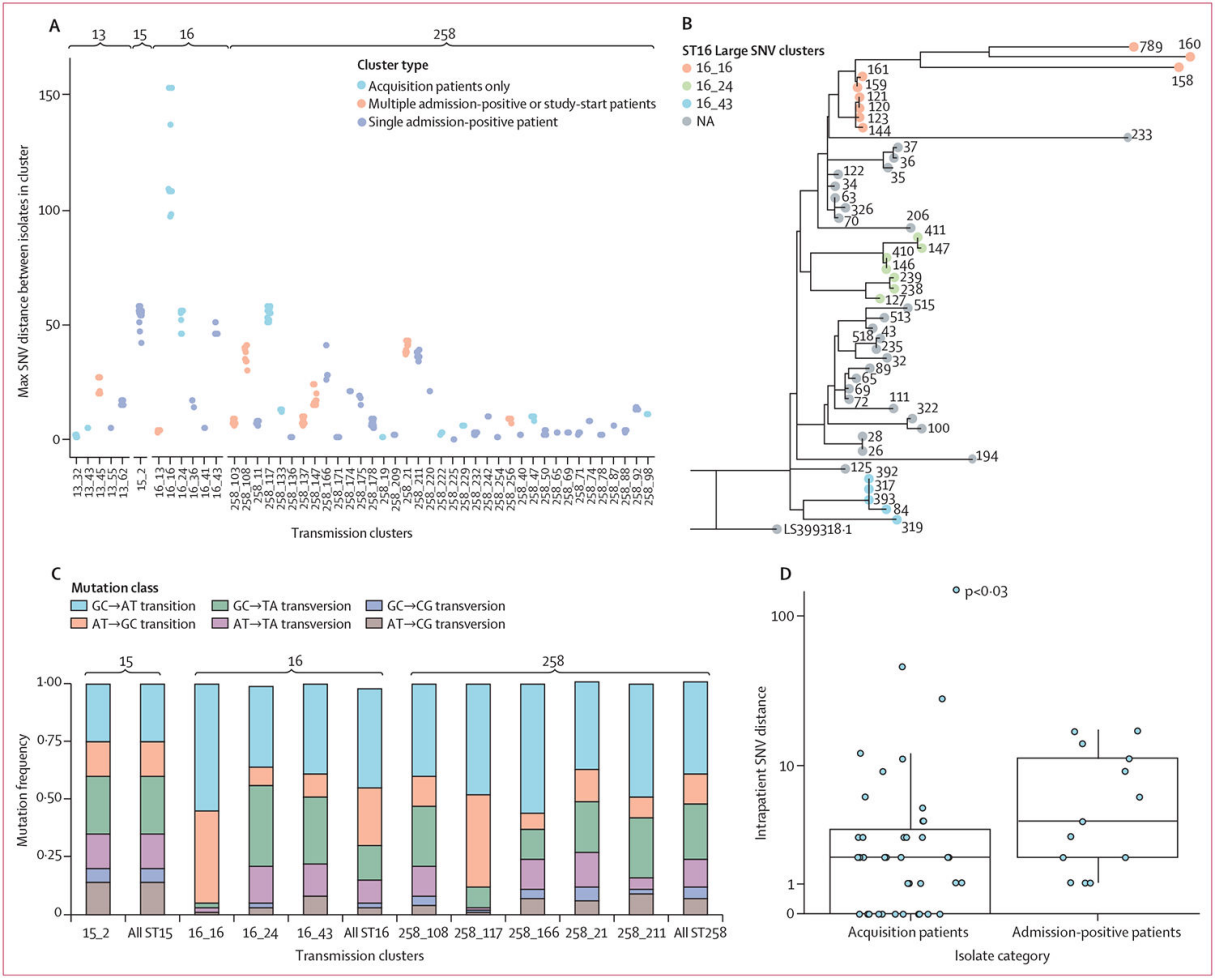
Genetic diversity in transmission clusters, prolonged colonisation, and emergence of hypermutator strains (A) Maximum pairwise SNV distance distinguishing isolates from the same cluster. Grey bars indicate the sequence type of the isolates in transmission clusters. (B) Phylogenetic tree indicating observed ST16 clusters with a pairwise genetic distance greater than 30 SNVs. (C) Observed frequencies in mutational classes across isolates included in each transmission cluster among clusters with a maximum pairwise SNV distance of at least 30 SNV. Bars on the right of each sequence-type group indicate the overall population frequency of mutational classes among members of that sequence type in the study population. (D) Maximum intrapatient, intracluster genetic diversity among admission-positive patients and acquisition patients. NA=not in one of the large SNV distance clusters. SNV=single nucleotide variant.

To take advantage of our threshold-free cluster detection, we explicitly characterised the range and variation of genetic diversity associated with putative intrafacility transmission. Calculation of the intracluster diversity showed that the maximum number of SNVs separating pairs of isolates in identified clusters ranged from zero to 153, with a median of nine SNVs. Although the majority of clusters varied by small genetic distances, nine (18%) of 49 clusters had larger SNV distances (>30 SNVs; figure 3A). One source of large SNV distances could be the improper inclusion of admission-positive patients who are not the true source of the transmission cluster. Indeed, for two of the nine clusters in which the intracluster genetic diversity was more than 30 SNVs (cluster 258_21 and cluster 258_108; figure 3A), two admission-positive patients were included, one of whom was genetically distant from other cluster members (appendix p 17). This finding suggests that, although genetic context was unable to discriminate between two putative source patients for these clusters, genetic distance was informative, suggesting that both context and distance can be valuable.

A second source of increased inter-patient SNV distances could be the accumulation of genetic variation during prolonged asymptomatic colonisation, and potential propagation of this variation via transmission. In support of this theory, we observed a distribution of intrapatient diversity among both admission-positive patients and patients who acquired KPC-*Kp* who contributed multiple isolates to a cluster (figure 3D). Moreover, we observed a significantly greater intrapatient diversity among patients who were positive for KPC-*Kp* at admisssion versus patients who acquired KPC-*Kp* (Wilcoxon rank-sum test, W=1680; p<0·03), supporting the role of prolonged colonisation driving intrapatient diversity (figure 3D).

In our examination of intrapatient diversity, we also observed several cases of extreme SNV distances between isolates of the same sequence type that were inconsistent with previously reported evolutionary rates for KPC-*Kp* (figure 3B; appendix p 17).^12,23^ We hypothesised that these large distances could be due to the emergence of hyper-mutator phenotypes, which has been reported for other commensal and pathogenic bacteria.^24^ Genomic signatures of hypermutators include specific mutational biases, as well as disruption of DNA mismatch repair genes, which can lead to a greater-than-expected number of mutations in a given time. Analyses of these genomic signatures showed that the transmission clusters with the largest numbers of intracluster SNVs (cluster 16_16, 153 SNVs; cluster 258_11, 58 SNVs; figure 3B-C; appendix p 17) had statistically significant skews in their mutational frequencies (exact multinomial test=lowF and trials=1 000 000; both tests p<0·05), supporting the role of mismatch repair mutations (eg, hypermutators), as well as a large insertion in *mutS* in isolates from one of the clusters (cluster 258_117; appendix p17).

Although the high KPC-*Kp* colonisation prevalence hindered the ability to perform contact tracing in the absence of genomic data, we hypothesised that, by examining patterns of patient overlap within clusters, we could gain insight into how KPC-*Kp* spread within the facility. We examined spatiotemporal overlap among cluster members during putative transmission windows (ie, between negative surveillance cultures and positive surveillance cultures) and found that, among the 129 acquisition events across the 49 clusters, 105 (81%), 85 (66%), and 19 (15%) of KPC-*Kp* acquisitions could be explained by overlap with another cluster member at the level of facility, floor, and room, respectively (appendix p 14). Compared with random groups of patients of the same size and patient type distribution (ie, numbers of admission-positive patients and patients who acquired KPC-*Kp*), actual transmission clusters were strongly enriched for these spatiotemporal overlaps among patients (permutation tests, p<0·001 for all locations; appendix p 14). In contrast to strong evidence for transmission between patients overlapping in space and time, we found little evidence for persistent environmental contamination as a source of transmission, with only 11 (9%) of the 129 acquisitions across clusters explained by sequential exposure to the facility, six (5%) by sequential exposure to a ward, and one (0·8%) by sequential exposure to a room (appendix p 14).

**Figure 4:**
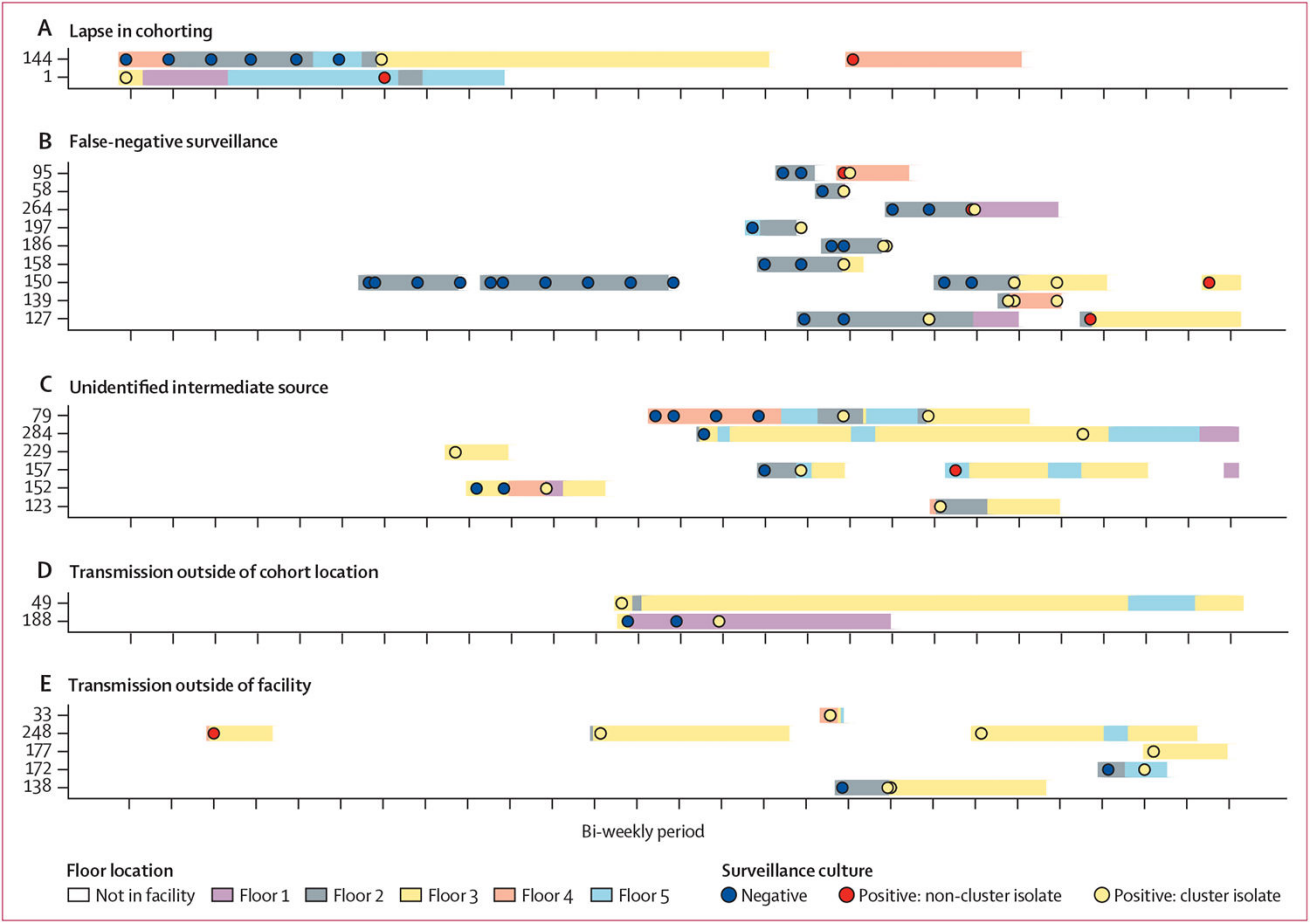
Descriptive vignettes from transmission clusters detected through the integration of genomic and surveillance data Patients are indicated on the y-axis and time is on the x-axis. Putative route of transmission within each cluster is indicated in the text above the cluster. Surveillance culturing information is indicated by the circles, and floor location in the LTACH is indicated by the coloured rectangles. (A) Transmission between admission-positive patient 1 to acquisition patient 144. Both patients were on the teal ward while patient 1 was positive and patient 144 was negative. (B) No admission-positive patient precedes several acquisition patients in this cluster; therefore, false-negative surveillance of a patient in the cluster or a patient not captured in the study is the probable source. (C) No spatiotemporal exposures between several patients indicate a missing intermediate source patient undetected by surveillance culturing. (D) Transmission between two patients who did not reside on the same ward indicates potential escape from cohort location, or transmission at a common location or via an unidentified common health-care worker source in the facility. (E) Multiple admission-positive patients and no spatiotemporal exposures in the LTACH indicates potential transmission outside of the facility before admission to the LTACH. All 49 transmission clusters detected are shown in the appendix (p 18). LTACH=long-term acute care hospital.

We sought to examine transmission clusters more holistically to gain insight into generalisable principles regarding KPC-*Kp* transmission pathways in the facility. Visual inspection of transmission clusters revealed several themes that manifested across multiple clusters (figure 4).

## Discussion

Harnessing WGS to optimise infection prevention is hindered by the absence of standardised criteria to detect transmission occurring within a health-care facility. In this study, we sought to dissect intrafacility transmission pathways in a high prevalence, endemic setting by leveraging a sample collection in which importation and acquisition events were comprehensively discerned via whole-hospital surveillance culturing for a 1-year period. This dense sampling allowed us to show the disadvantages associated with imposing an SNV threshold to delineate transmission within a facility, and then to apply a threshold-free approach to group acquisition events into transmission clusters traced back to importation events. Examination of these threshold-free transmission clusters yielded insight into the genetic diversity underlying true transmission pairs and allowed for deconvolution of transmission pathways in a setting with extremely high colonisation prevalence.

Our unbiased view of the genetic diversity underlying putative transmission events showed how the imposition of a strict SNV threshold could lead to significant false-positive and false-negative transmission inferences. The primary source of false-negative inferences is the accumulation of genetic variation during prolonged asymptomatic colonisation, which can manifest in large and uneven genetic distances between patients linked by transmission within the facility.^13,16,17^ Moreover, we were able to show that this intrapatient diversity can be further amplified by the emergence of hypermutator strains, which yield genetic distances that would elude any approach grounded in SNV thresholds.^9,12^ Conversely, SNV threshold approaches can also lead to false-positive transmission inferences due to the importation of closely related strains that might be linked by recent transmission at a connected health-care facility.^12,14,25,26^ This finding is consistent with many studies that have shown that the transfer of colonised patients across highly connected health-care networks can lead to closely related strains in different facilities,^6,9,12,14^ making it challenging to discern where recent transmission events occurred. However, despite disadvantages in the sole reliance on SNV thresholds, we observed cases where consideration of both genetic context and distance together allowed for a more precise attribution of the acquisition source. Moreover, in low-prevalence settings where strain importation is rare, small SNV thresholds are likely to be accurate predictors of intrafacility transmission.

Our study has several limitations related to biases in sampling. First, although we sampled 94% of patients in the facility during the 1-year period, only a single or small number of colonies (representative unique morphologies) were collected and sequenced per patient, and patients were not resurveilled systematically once identified to carry KPC-*Kp*. Therefore, we might have missed cases where a patient imported multiple strains into the facility, where a patient acquired a second KPC-*Kp* strain later in their hospital stay, or where horizontal transfer of bla_KPC_ occurred within a patient. Any of these limitations could potentially account for some of the cases for which we were unable to identify a cluster-source patient. Second, our lack of knowledge of where patients were before hospital admission prevents us from understanding how transmission at connected health-care facilities (or at the current facility before the start of the study) influenced grouping of patients in clusters. We hypothesise that these transmission events outside of the facility accounted for cases where patients who were positive for KPC-*Kp* at admission were not the first members of their cluster to test positive. Third, there were nine patients who were not cultured until after 3 days of being in the facility, hindering our ability to know whether they were colonised on admission. Due to the high colonisation pressure and a desire not to split transmission clusters, we assumed that these individuals had acquired KPC-*Kp* in the facility. Fourth, there is an inherent limit of detection of surveillance culturing, which is probably associated with variation in the density of KPC-*Kp* colonisation in the gut.^27^ These cryptic cases could account for clusters without patients who were positive for KPC-*Kp* at admission or missing spatiotemporal exposures between patients, and could account for some cases of large intracluster genetic variation. Last, we did not perform environmental culturing and could therefore have missed persistently colonised equipment or procedure rooms as sources of transmission, although we expect that the groups of patients in clusters would remain consistent despite a missing environmental intermediate source.^28^

Overall, our results highlight the potential for WGS to improve infection surveillance and prevention when combined with appropriate sampling and analytical strategies that are jointly tailored to generate actionable hypotheses. The SNV threshold-free approach applied could be implemented with only admission and discharge surveillance culturing, although higher resolution sampling would facilitate more rapid detection and precise delineation of transmission pathways within clusters. Importantly, by relying on shared variants, inferences should be robust to the specific species or strain, thereby circumventing the need for constant refinement of discriminatory criteria and facilitating clearer interpretation and more effective intervention by health-care epidemiologists. Future studies are needed to confirm these hypotheses, and to continue to rigorously evaluate methodologies for transmission inference across pathogens with different characteristics and health-care settings of varying complexity.

## Supplementary Material

1

## Figures and Tables

**Table:** Epidemiological categorisation of transmission clusters

	Description	Number of clusters (N=49)*
Patient-to-patient transmission	Importation by admission-positive or study-start patient and spatiotemporal overlap explanations for all acquisitions	24 (49%)
Missing intermediate source	No spatiotemporal exposure explanation for at least one acquisition	11 (22%)
Multiply colonised admission-positive patient	Admission-positive or study-start patient is the first to test positive for an isolate that is not included in the cluster, and spatiotemporal overlap explanations for all except one acquisition; one acquisition without overlap is permitted to account for failure to capture the true source isolate on the admission-positive patient′s first culture	5 (10%)
False-negative surveillance	Admission-positive or study-start patient is not the first patient to test positive in the cluster, but all but one acquisition has spatiotemporal exposure explanations; one acquisition without overlap is permitted to account for false-negative surveillance	7 (14%)
Missing source	Clusters with unclear source that do not fit into other transmission cluster categories	2 (4%)

* All transmission clusters are shown in the appendix (p 18).

## Data Availability

Sequence data and limited meta-data are available under National Center for Biotechnology information BioProject PRJNA603790.^29^

## Research in context

### Evidence before this study

We searched PubMed for studies published in English before May 1, 2021, with no start date restriction, with the search terms “transmission” AND “whole-genome” AND ”(snp OR snv)” AND “(cut-off OR threshold) NOT (SARS-CoV-2 OR virus or HIV)”. We identified 18 reports that used whole genome sequencing to study transmission, primarily in health-care settings. Several of these studies attempted to identify optimal single nucleotide variant (SNV) cutoffs for delineating transmission. These studies were largely limited to either clinical isolate collections or active surveillance targeted to a handful of hospital units. Even when the same species was considered in different studies, different optimal SNV thresholds were reported.

### Added value of this study

To understand the limitations of an SNV threshold approach for tracking transmission, we leveraged a dataset that comprised admission and every-other-week *KPC-Kp* surveillance culturing for every patient entering a hospital over the course of 1 year. By performing genomic analysis of 435 isolates from the 256 patients colonised with *KPC-Kp*, we systematically showed disadvantages of the use of SNV thresholds for transmission inference that stem from the importation of closely related strains from connected health-care facilities, variation in genetic heterogeneity of colonising populations, and uneven evolutionary rates of *KPC-Kp* strains colonising patients. We implemented an alternative approach for tracking transmission in health-care facilities that relies on genetic context—instead of genetic distance—to group patients into intrafacility transmission clusters. We applied this approach to our *KPC-Kp* genomes and showed that the resultant transmission clusters are strongly enriched in patients with spatiotemporal overlap, and that clusters can be interrogated to identify putative targets to interrupt transmission.

### Implications of all the available evidence

Advances in the speed and decreased cost of genome sequencing are making it increasingly feasible to perform routine sequencing to track transmission in health-care settings. However, a crucial barrier to these efforts is that there are no clear criteria for inferring transmission that generalises to diverse strains of health-care pathogens and that are robust to variation in organism prevalence and differences in connectivity of local health-care networks. In this study, we show that by combining genome sequencing with surveillance data, health-care transmission can be inferred in a threshold-free manner. The success of this approach in a setting with high importation and transmission rates bodes well for its generalisability to track transmission for other health-care pathogens in similarly challenging settings.
