# Supplementary material for: Threshold-free genomic cluster detection to track transmission pathways in health-care settings: a genomic epidemiology analysis
Source: Lancet Microbe. Author manuscript; Available in PMC 2023 Jan 23. (PMC9869340; doi:10.1016/S2666-5247(22)00115-X)
Supplement: 1 [file NIHMS1834109-supplement-1.pdf]

THE LANCET
Microbe

Supplementary appendix

This appendix formed part of the original submission and has been peer reviewed. We post it as supplied by the authors.

Supplement to: Hawken SE, Yelin RD, Lolans K, et al. Threshold-free genomic cluster detection to track transmission pathways in health-care settings: a genomic epidemiology analysis. *Lancet Microbe* 2022; published online July 5. https://doi.org/10.1016/S2666-5247(22)00115-X.

Table of Contents

Supplementary Methods *1*

Details on Parent Intervention Study 1

Definition of isolates as imported or acquired in the facility 1

Whole genome sequencing & genome processing 2

Bioinformatics analysis 2

Threshold-free transmission cluster detection 3

Patient overlap analysis 5

Supplementary references 6

Supplementary figures and tables *8*

Table S1: Distribution of KPC-Kp strains isolated from colonized LTACH patients. 8

Table S2: Reference genomes for MLST-specific Alignments 9

Table S3. Clustering by SNV threshold, percentage of acquisition isolates and patients from threshold-free clusters linked to at least one isolate from an index patient in the threshold-free clusters. 10

Figure S1. Endemicity of KPC-Kp in the LTACH is due to extensive importation and acquisition of multiple KPC-Kp strains throughout the study. 11

Figure S2. Complexity of discerning KPC-Kp transmission chains in the LTACH is illustrated by extensive importation and acquisition of multiple strains and patients with shared time in the LTACH. 12

Figure S3: Schematic of genomic transmission cluster detection strategy that integrates shared variants from whole-genome sequences with surveillance data. 13

Figure S4: Epidemiologic exposures within transmission clusters point to frequent acquisition of an isolate from outside of a patient's room location (i.e., ward or facility) and infrequent acquisition linked to sequential occupation of same room, ward, or facility 14

Figure S5: Uncertainty of index patient source in clusters defined by SNV thresholds compared to threshold-free cluster detection. 15

Figure S6: Phylogenies for each MLST indicating threshold-free clusters with higher than expected genetic variation. (SNV distances >30) 17

Figure S7: Patient bed traces and surveillance information for the 49 transmission clusters detected through the integration of genomic and surveillance data. 18

Supplementary Methods

Details on Parent Intervention Study

Detailed information regarding the study design, intervention bundle and data collection are available in Hayden et. al 2015[1]. Briefly, the intervention included: i) rectal surveillance swab culture-based screening using the direct ertapenem disk method followed by PCR confirmation of blaKPC,[1,2] of all LTACH patients at LTACH admission and every two weeks thereafter until a patient received a positive test (94% adherence), ii) physical separation of KPC-Kp-positive and KPC-Kp-negative patients by placing KPC-Kp-positive patients in ward cohorts (91% adherence), iii) daily chlorhexidine bathing of all patients in the LTACH and iv) a hand hygiene campaign.[1–4] Extensive detail regarding microbiological methods in BSL-2 conditions, study design and intervention are included in prior manuscripts.[1,2]

Definition of isolates as imported or acquired in the facility

Patients were grouped into categories based on surveillance culture results. Patients who were either positive at the start of the study or within three days of LTACH admission were considered potential sources of KPC-Kp importation and onward transmission within the LTACH. Patients who were KPC-Kp-negative on their first surveillance culture, and then KPC-Kp-positive after day three of admission, were assumed to have acquired KPC-Kp in the facility. If a patient's first surveillance sample was collected more than three days after admission and was positive for KPC-Kp, the patient was also assumed to have acquired KPC-Kp in the facility for the purposes of the transmission cluster detection algorithm. When an admission-positive patient acquired an additional KPC-Kp strain (as evidenced by multi-locus sequence type

(MLST) inferred from WGS data) during their stay this was termed a secondary acquisition, and such isolates from admission positive patients were eligible to be included as acquisition isolates for transmission cluster detection.[4]

Whole genome sequencing & genome processing

Glycerol stocks containing unique morphologies of KPC-Kp isolates were stored at -80°C prior to cultivation on LB agar for DNA isolation.[1,2] DNA was extracted with the MoBio PowerMag Microbial DNA kit and prepared for sequencing on an Illumina MiSeq or HiSeq instruments using the NEBNext Ultra kit and sample-specific barcoding. Library preparation and Illumina sequencing were performed at the Center for Microbial Systems at the University of Michigan and the University of Michigan Sequencing Core. Genomes were sequenced on the with 250bp paired end reads on the MiSeq and 125 bp paired end reads on the HiSeq 2500. Quality of reads was assessed with FastQC version 0.11.9,[5] and Trimmomatic version 0.39[6] was used for trimming adapter sequences and low-quality bases (parameters - seed_mismatches: 2, palindrome_clipthreshold: 30, simple_clipthreshold: 10, minadapterlength: 8, SLIDINGWINDOW:4:20 , MINLEN:40). In total, 462 isolates were sequenced, with the 435 isolates from 256 unique patients passing QC being used in downstream analyses.

Bioinformatics analysis

Genomes were assigned STs using the Ariba 2.14.4[7] and the Pasteur database accessed in February 2020 (https://bigsdb.pasteur.fr/klebsiella/). SNV calling was performed as in Han *et al*.[8]

The variant calling pipeline can be found at https://github.com/Snitkin-Lab-Umich/variant_calling_pipeline. To summarize, raw reads were mapped to the ST specific reference genomes listed in **Supplementary Table 2** using BWA-MEM algorithm from BWA-0.7.17 (r1188)[9]. Variant calling was performed with samtools 1.11.[10,11] Reference genomes with matching sequence types (STs) to study strains were chosen to maximize core-genome orthologous regions for SNV identification and decrease erroneous variant calls[12]. Consensus alignments generated from read mapping to ST specific reference genomes underwent detection and masking of putative recombinant regions using gubbins[13]. Lastly, reference genome annotations were generated using Prokka[14] version 1.14.5 and variant annotations were predicted using snpEff version 4.3t[15].

Recombination filtered variant alignments containing core and non-core variant positions were used: i) to generate pairwise (genome by genome) single-nucleotide variant (SNV) distance matrices and shared-variant matrices, ii) interrogate mutation class frequencies and iii) construct maximum parsimony phylogenetic trees used for transmission cluster identification (See Procedures). PanIsa 0.1.4 was used to detect insertion sequences in bam files containing WGS alignments.[16] All whole-genome sequence analyses were performed in R version 3.6.1.[17]

Threshold-free transmission cluster detection

Transmission cluster detection using a SNV threshold-free approach was performed on isolates from sequence types (STs) that were present in at least two patients including at least one acquisition patient (**Table S1**). ST-specific recombination filtered variant alignments were used to create maximum parsimony phylogenetic trees using the optim.parsimony function in version 2.7.1 of the phangorn package in R, which formed the basis for cluster detection. The rationale

for using a maximum parsimony algorithm for tree construction was that given the small number of variants distinguishing endemic strains, we wanted to treat any variant as an equally informative discriminatory marker with which to group isolates into transmission clusters. To increase the resolution of cluster detection we also included positions where one or more sequences were recombination masked or had an insertion/deletion relative to the reference. Maximum parsimony trees were then rooted using the external reference genome (**Table S2**). Transmission clusters were identified as monophyletic clades in the tree as follows. First, all clades in the tree were identified using the subtrees function in version 5.5 of the ape package in R. Second, clades were evaluated to determine whether they represented valid transmission clusters. To be a valid cluster the following criteria had to be met: i) all members of the cluster must share at least one variant not present in non-cluster members (i.e. possess a cluster defining variant), ii) multiple admission positive patients who could have started the cluster (i.e. were positive on or before the date of all acquisition patients) were only allowed if none uniquely shared variants with cluster members (i.e. didn't share a more recent common ancestor with cluster members) and iii) the cluster does not contain a valid cluster within it (i.e. there is not a clade that is a subset of the current one that is also a valid cluster based on the first two criteria). Finally, each sequence was assigned to the valid cluster that contained the largest number of sequences from an admission positive patient that could have started the cluster or from acquisition patients. This algorithm has the benefit of prioritizing assignment of acquisition patients to clusters where there exists an admission positive patient who could have started the cluster, but also allows for clusters to exist that do not have an admission positive patient so long as the other criteria are met.

Patient overlap analysis

Location data were abstracted from patient bed traces, i.e. patient bed and room location(s) over time. Spatiotemporal overlap explanations for cross-transmission between patients in clusters were defined as patients being in the same location (e.g. facility, ward or room) at the same time during the period between when a putative donor patient in the cluster was last negative for the isolate up until and including the day the recipient tested positive for the isolate. The last-negative date was chosen as a conservative bound for the earliest time acquisition could have occurred in order to account for acquisitions occurring between biweekly sampling dates. Sequential exposure was evaluated for the same timeframe, but restricted to patients in the same location separated by time, where the putative donor had been in a location first and the recipient later occupied the same location in the window between their transition from negative to positive surveillance, and no spatiotemporal exposure between donors in the cluster and the recipient could explain the recipients' acquisition.

Supplementary references

1. Hayden, M. K. *et al*. Prevention of Colonization and Infection by Klebsiella pneumoniae Carbapenemase–Producing Enterobacteriaceae in Long-term Acute-Care Hospitals. *Clin. Infect. Dis.* **60**, 1153–1161 (2015).
2. Lolans, K., Calvert, K., Won, S., Clark, J. & Hayden, M. K. Direct Ertapenem Disk Screening Method for Identification of KPC-Producing Klebsiella pneumoniae and Escherichia coli in Surveillance Swab Specimens. *J. Clin. Microbiol.* **48**, 836–841 (2010).
3. Haverkate, M. R. *et al*. Modeling Spread of KPC-Producing Bacteria in Long-Term Acute Care Hospitals in the Chicago Region, USA. *Infect. Control Hosp. Epidemiol.* **36**, 1148–1154 (2015).
4. Hawken, S. E. *et al*. Cohorting KPC+ Klebsiella pneumoniae (KPC-Kp)-positive patients: A genomic exposé of cross-colonization hazards in a long-term acute-care hospital (LTACH). *Infect. Control Hosp. Epidemiol.* **41**, 1162–1168 (2020).
5. Babraham Bioinformatics - FastQC A Quality Control tool for High Throughput Sequence Data. https://www.bioinformatics.babraham.ac.uk/projects/fastqc/.
6. Bolger, A. M., Lohse, M. & Usadel, B. Trimmomatic: a flexible trimmer for Illumina sequence data. *Bioinformatics* **30**, 2114–2120 (2014).
7. Hunt, M. *et al*. ARIBA: rapid antimicrobial resistance genotyping directly from sequencing reads. *Microb. Genomics* **3**, e000131 (2017).
8. Han, J. H. *et al*. Whole-Genome Sequencing To Identify Drivers of Carbapenem-Resistant Klebsiella pneumoniae Transmission within and between Regional Long-Term Acute-Care Hospitals. *Antimicrob. Agents Chemother.* **63**, e01622-19 (2019).

9. Li, H. & Durbin, R. Fast and accurate short read alignment with Burrows-Wheeler transform. *Bioinforma. Oxf. Engl.* **25**, 1754–1760 (2009).

10. Li, H. *et al.* The Sequence Alignment/Map format and SAMtools. *Bioinformatics* **25**, 2078–2079 (2009).

11. Li, H. & Durbin, R. Fast and accurate short read alignment with Burrows–Wheeler transform. *Bioinformatics* **25**, 1754–1760 (2009).

12. Bush, S. J. *et al.* Genomic diversity affects the accuracy of bacterial single-nucleotide polymorphism-calling pipelines. *GigaScience* **9**, giaa007 (2020).

13. Croucher, N. J. *et al.* Rapid phylogenetic analysis of large samples of recombinant bacterial whole genome sequences using Gubbins. *Nucleic Acids Res.* **43**, e15 (2015).

14. Seemann, T. Prokka: rapid prokaryotic genome annotation. *Bioinforma. Oxf. Engl.* **30**, 2068–2069 (2014).

15. Cingolani, P. *et al.* A program for annotating and predicting the effects of single nucleotide polymorphisms, SnpEff: SNPs in the genome of Drosophila melanogaster strain w1118; iso-2; iso-3. *Fly (Austin)* **6**, 80–92 (2012).

16. Treepong, P. *et al.* panISa: ab initio detection of insertion sequences in bacterial genomes from short read sequence data. *Bioinformatics* **34**, 3795–3800 (2018).

17. R, C. T. *R: A language and environment for statistical computing*. (R Foundation for Statistical Computing, 2019).

18. Pathogenwatch | Genomes. https://pathogen.watch/genomes/all?genusId=570&speciesId=573.

Supplementary figures and tables

Table S1: Distribution of KPC-Kp strains isolated from colonized LTACH patients.

MLST§§	†Number of isolates	Number of patients	Number of admission positive/study-start isolates	Number of patients with admission positive/study-start isolates	Number of acquisition isolates	Number of acquisition patients	Number of admission positive patients with acquisitions occurring afterwards	Number of acquisition patients potentially acquiring colonization from a plausible source	*Potential cross-transmission link in the LTACH
13	63	37	25	18	38	22	18	15	yes
14	7	5	2	1	5	4	1	2	yes
15	17	11	1	1	16	10	1	7	yes
16	47	32	15	15	32	20	15	17	yes
20	6	6	1	1	5	5	1	4	yes
36	1	1	1	1	0	0	0	0	no
134	1	1	1	1	0	0	0	0	no
193	2	2	2	2	0	0	0	0	no
258	271	177	101	83	170	104	83	86	yes
327	6	4	0	0	6	4	0	0	yes
834	2	1	2	1	0	0	0	0	no
874	7	5	4	3	3	2	2	1	yes
883	1	1	1	1	0	0	0	0	no
950	3	1	0	0	3	1	0	0	no
5890	1	1	1	1	0	0	0	0	no
Total‡	435	285	157	129	278	172	121	132	

*Potential cross-transmission link in LTACH during study inferred by at least two patients with isolate of the same MLST and at least one patient acquiring colonization with an isolate of the same MLST.
†Isolate total represents isolates with quality WGS data; 27 samples were excluded from the 462 total isolates obtained due to poor sequence quality.
‡Totals do not add to the number of patients (N=256) because patients had >1 isolate represented by different MLSTs.
§§ Prevalence of all *K pneumoniae* of STs identified in this study (with the exception of ST 5890, which was identified in this study), can be found on the *Klebsiella pneumoniae* page of Pathogen watch. (https://pathogen.watch/genomes/all?genusId=570&speciesId=573)[18]

Table S2: Reference genomes for MLST-specific Alignments

*In the case where no high-quality MLST-specific assembly was available in NCBI databases, KPNIH1 (ST 258) was used as the reference genome.

MLST	Reference Genome
13	CP014123
14	CP014004
15	CP015990.1
16	LS399318.1
20*	KPNIH1
258*	KPNIH1
874*	KPNIH1

Table S3. Clustering by SNV threshold, percentage of acquisition isolates and patients from threshold-free clusters linked to at least one isolate from an index patient in the threshold-free clusters.

**Clustering method**	Acquisition patients linked to same importation patient as in threshold-free cluster (N=64)	Acquisition patients in threshold-free clusters (N=100) linked to 0 importation patients	Acquisition patients in threshold-free clusters (N=100) linked to 1 importation patients	Acquisition patients in threshold-free clusters (N=100) linked to >1 importation patients
**10 SNV**	43	56	31	13
**20 SNV**	48	39	25	36
**Threshold-free**	64	36	47	17

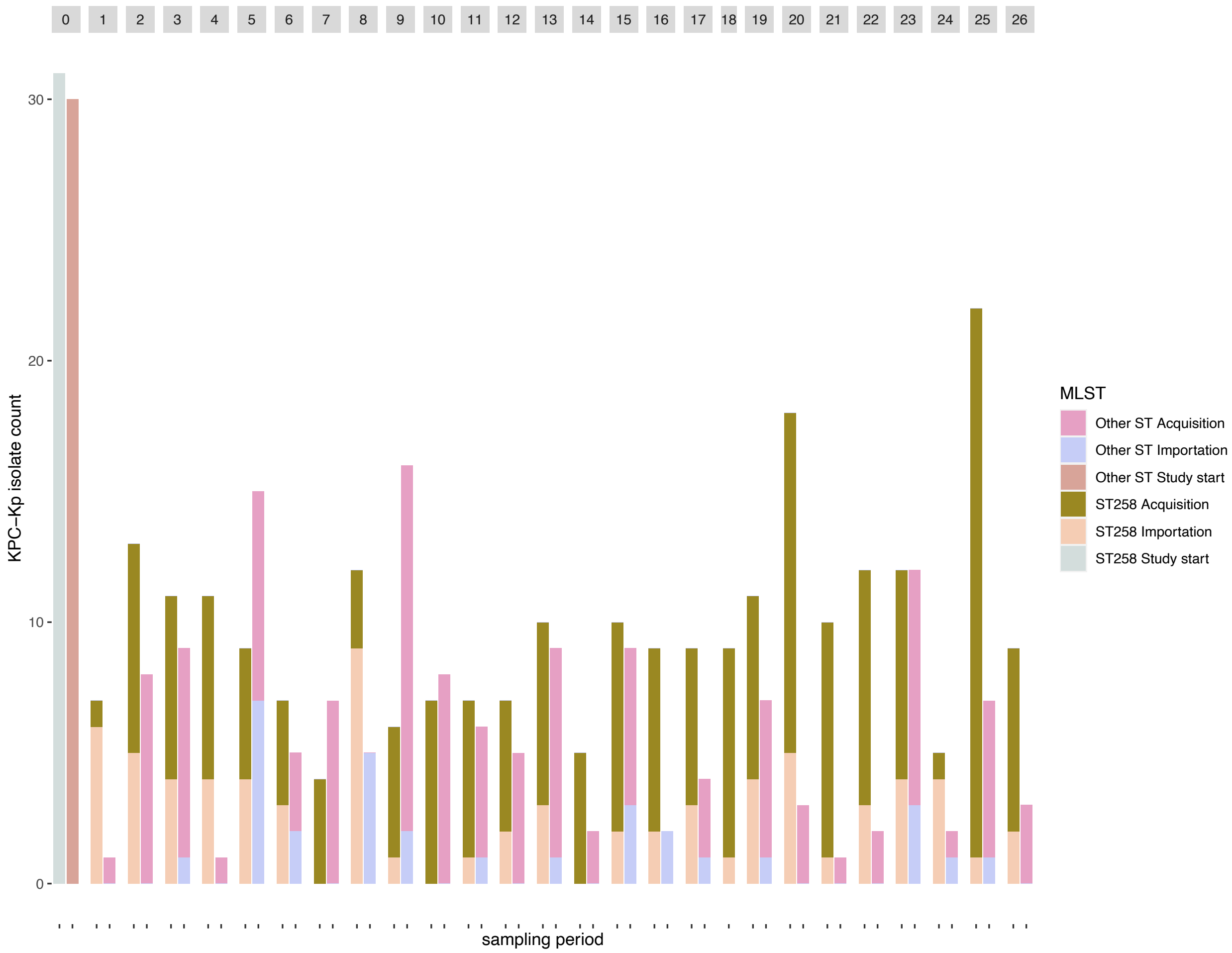

Figure S1. Endemicity of KPC-Kp in the LTACH is due to extensive importation and acquisition of multiple KPC-Kp strains throughout the study.

MLST of isolates obtained through bi-weekly rectal surveillance culturing of LTACH patients. Grey boxes indicate the study start (time 0) and 14-day surveillance periods throughout the study. Bars indicate the KPC-Kp isolates collected at the beginning of the study, after importation or acquisition (>3 days after a patient was in the LTACH). Legend indicates ST258 or other MLST of isolates.

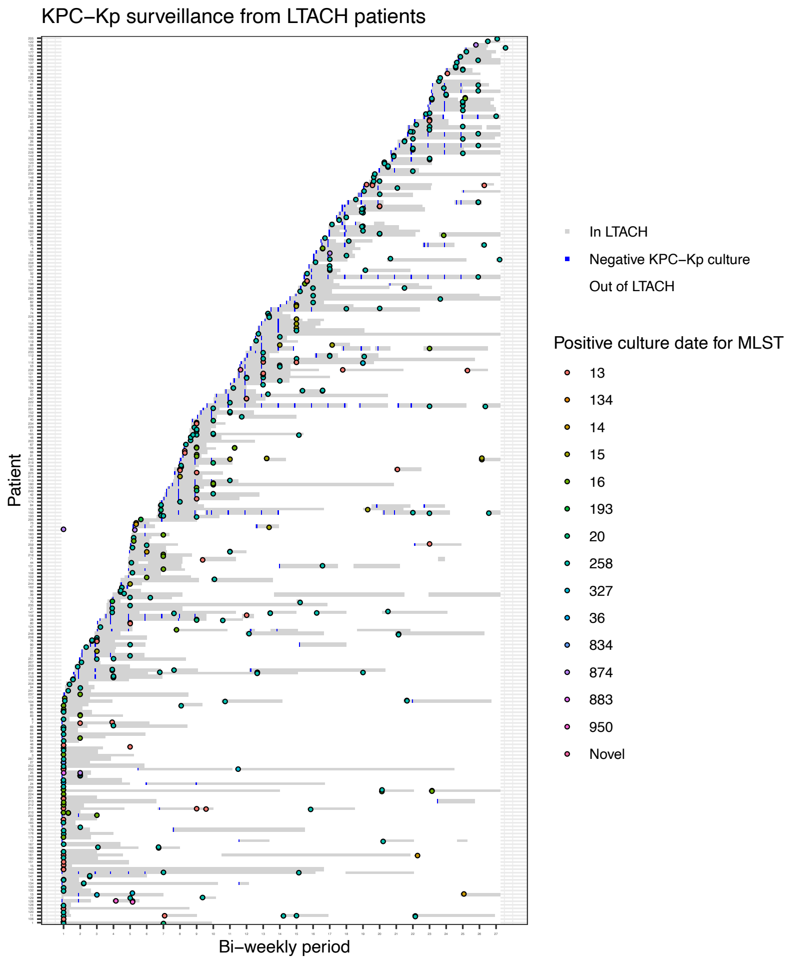

Figure S2. Complexity of discerning KPC-Kp transmission chains in the LTACH is illustrated by extensive importation and acquisition of multiple strains and patients with shared time in the LTACH.

A. Patient bed trace showing surveillance of patients who either imported or acquired KPC-Kp colonization in the LTACH. The order of patients on the Y-axis is by first date in the LTACH. Grey bars indicate patients in the LTACH, white indicates outside of the facility, colored circles indicate the MLSTs of isolates obtained by positive surveillance cultures from a patient on a collection date (x axis).

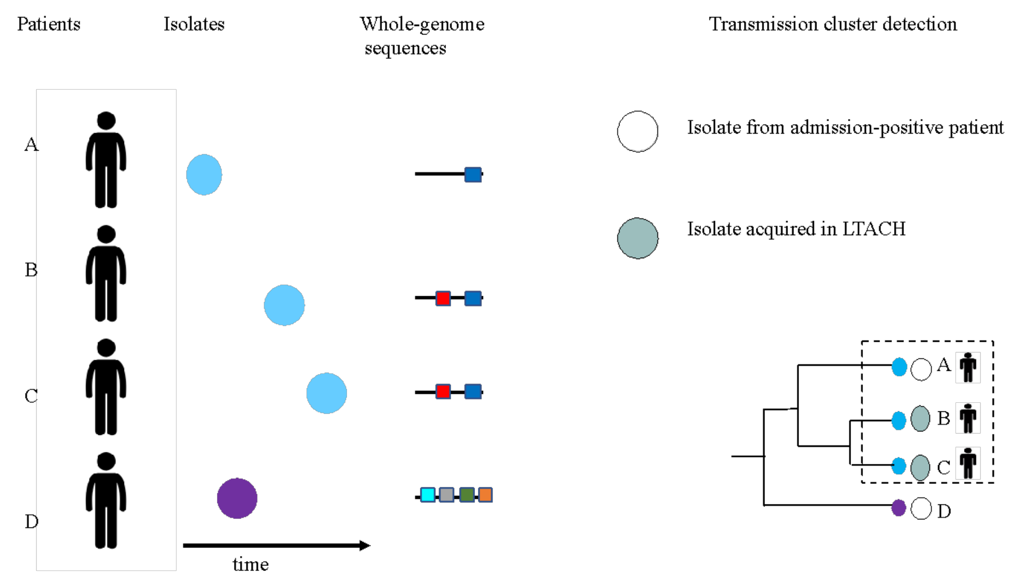

Figure S3: Schematic of genomic transmission cluster detection strategy that integrates shared variants from whole-genome sequences with surveillance data.

Shared variants in whole genome sequences (black lines, variants are colored boxes) from isolates sampled from patients are used to construct a maximum parsimony phylogeny. Transmission clusters are defined by the maximum subtree in the phylogeny that contains isolates from a single admission-positive patient who imported the isolate from outside the facility. Valid transmission clusters must contain at least a single unique variant (blue box) that distinguishes cluster from non-cluster isolates (A, B and C isolates (dashed box) vs isolate D), and at least two patients including at least one acquisition patient who acquired KPC-Kp colonization in the LTACH. Clusters with multiple admission positive/study-start patients are valid if the isolates share unique variants with other cluster members. Clusters with no admission positive patients are valid if there existed no subtree that contained an admission positive isolate.

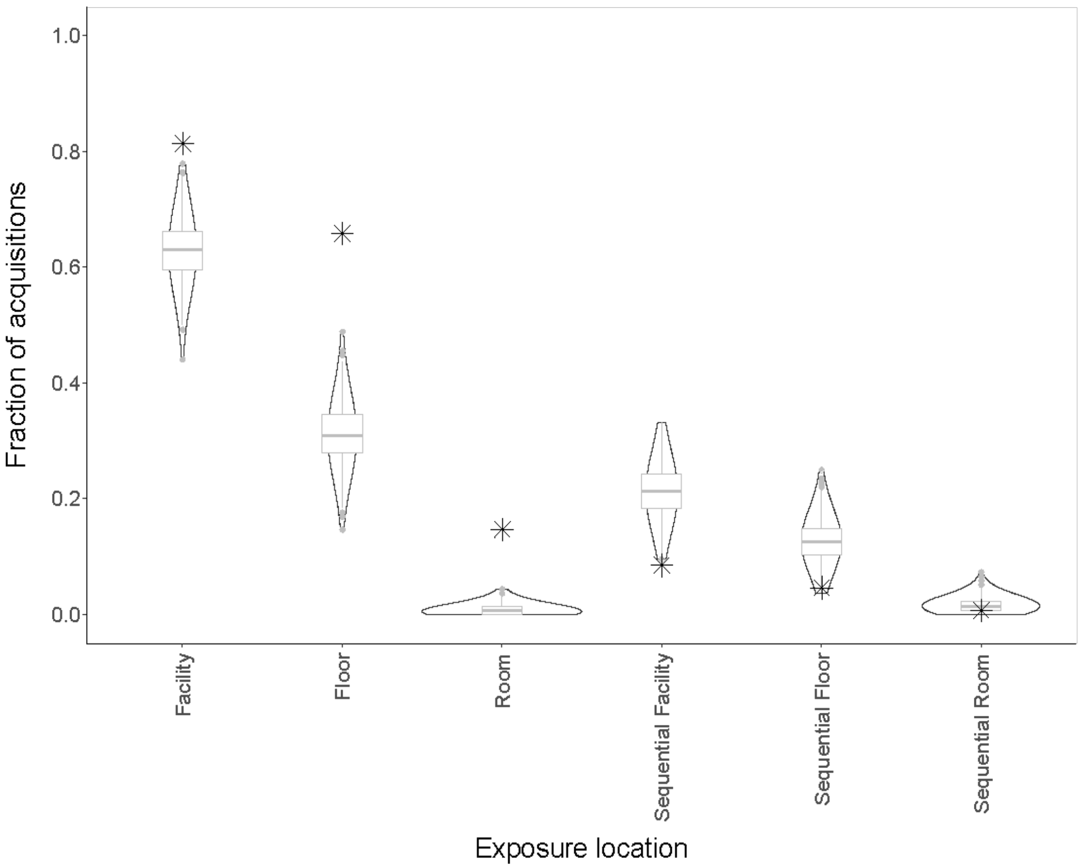

Figure S4: Epidemiologic exposures within transmission clusters point to frequent acquisition of an isolate from outside of a patient's room location (i.e., ward or facility) and infrequent acquisition linked to sequential occupation of same room, ward, or facility.

X-axis indicates locations, Y axis indicates fraction of acquisitions in transmission clusters that could be attributed to putative donor and recipient (acquisition) patients in the cluster being in the same place at the same time (spatiotemporal exposure) or in the same place separated by time after a donor had left that location (sequential exposure). Stars indicate observed values, violins indicate exposures among permuted random transmission clusters. Spatiotemporal exposure is enriched in transmission clusters compared to permuted groups of patients of the same size and patient makeup (admission positive and acquisition patients) as the observed clusters (permutation tests, $p<0.001$ for all locations). Sequential exposure is not enriched in transmission clusters compared to random clusters (permutation tests, $p>0.60$ for all locations.)

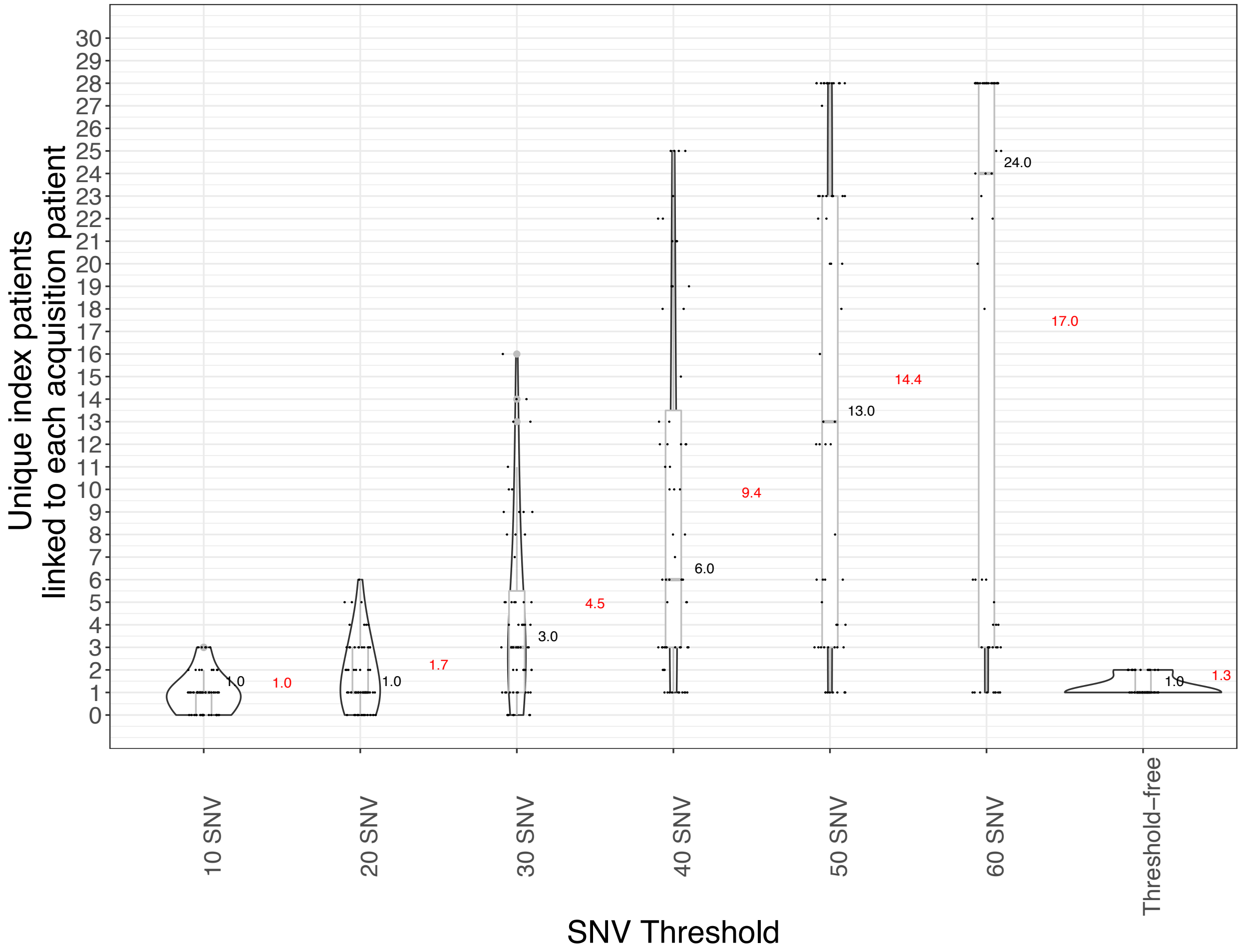

Figure S5: Uncertainty of index patient source in clusters defined by SNV thresholds compared to threshold-free cluster detection.

X-axis indicates SNV threshold, Y-axis indicates number of unique admission positive patients linked to each acquisition patient who is included in threshold-free clusters that included at least 1 admission positive patient. At each threshold, violin and box plots indicate the distribution of the number of patients (text: red=mean, black=median number of index patients linked to each acquisition patient)

15

phylogeny

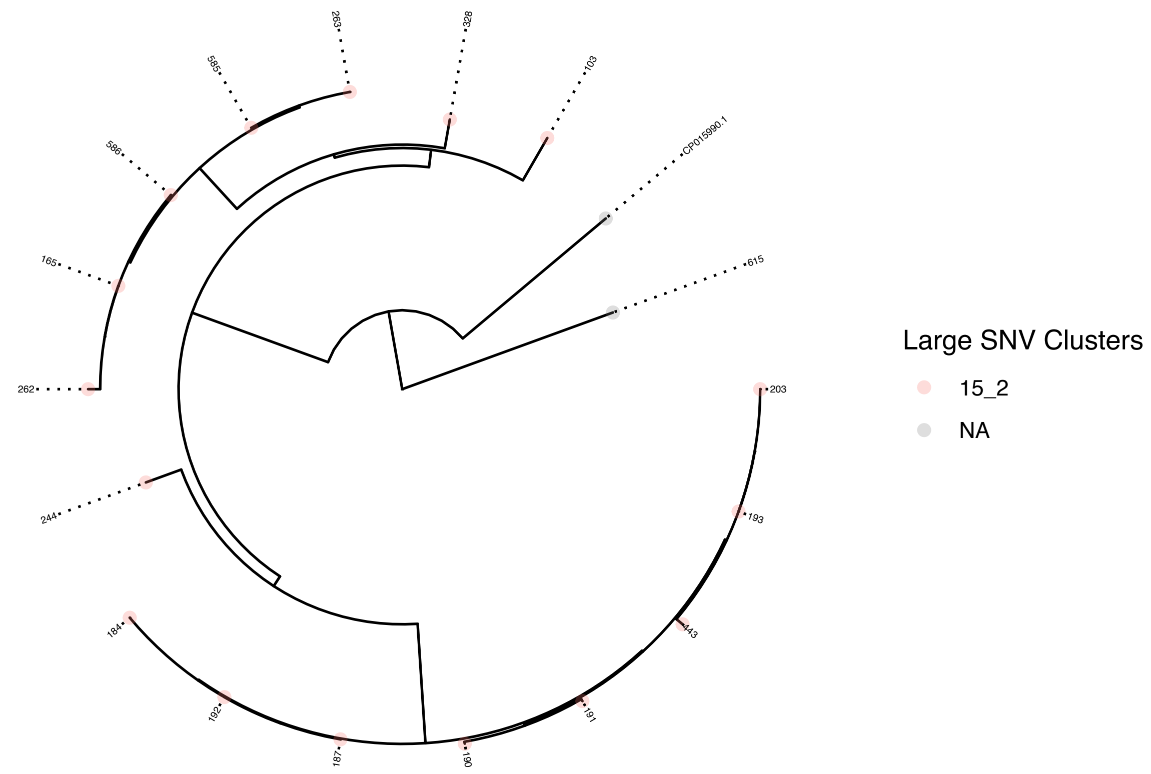

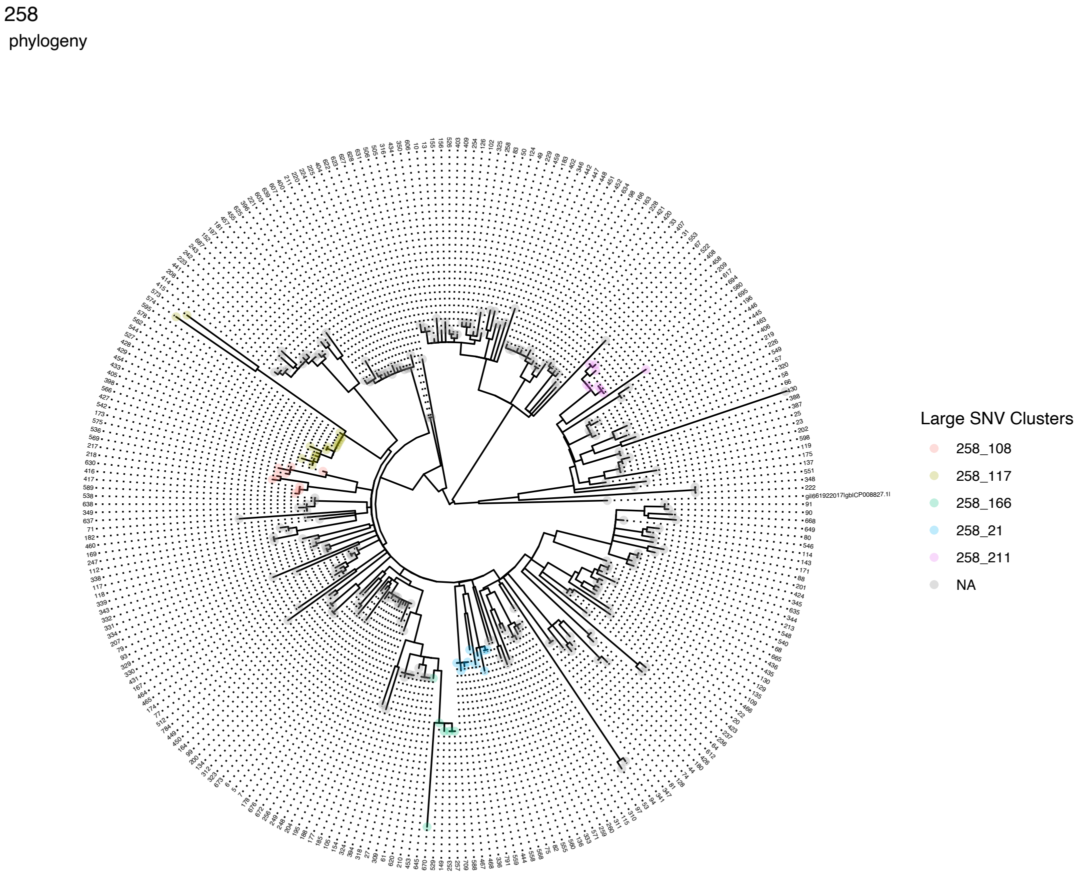

Figure S6: Phylogenies for each MLST indicating threshold-free clusters with higher than expected genetic variation. (SNV distances >30).

Potential mutator clusters are highlighted by the colors indicated in the legend. The ST16 phylogeny is in the main manuscript text.

Figure S7: Patient bed traces and surveillance information for the 49 transmission clusters detected through the integration of genomic and surveillance data.

Patients are indicated on the Y axis and time is on the X -axis.

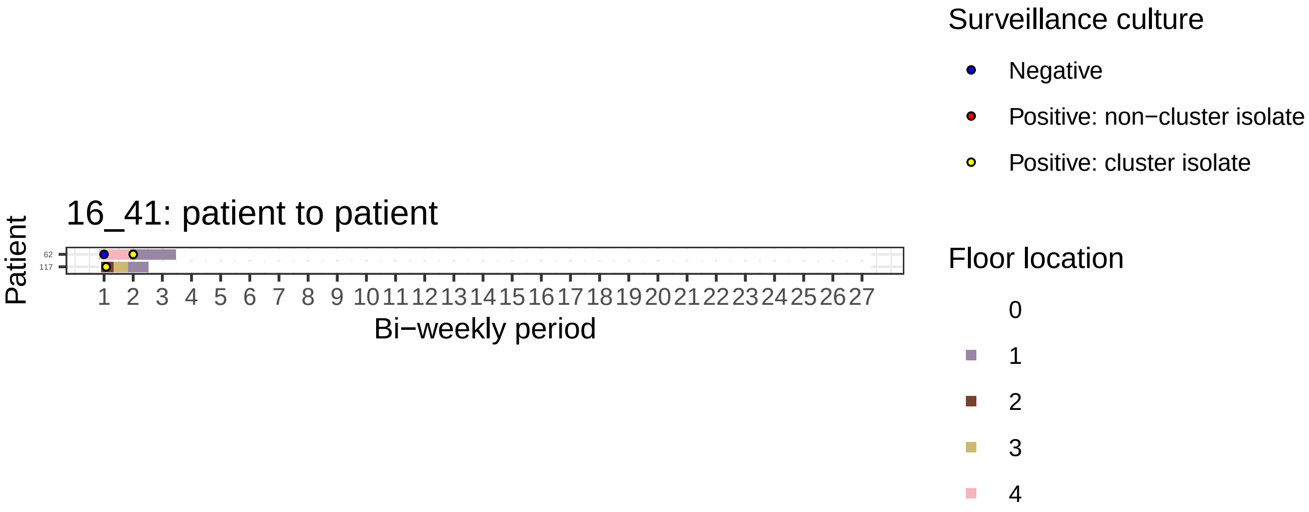
16_41: patient to patient
Patient
62
117
1 2 3 4 5 6 7 8 9 10 11 12 13 14 15 16 17 18 19 20 21 22 23 24 25 26 27
Bi−weekly period
Surveillance culture
Negative
Positive: non−cluster isolate
Positive: cluster isolate
Floor location
0
1
2
3
4

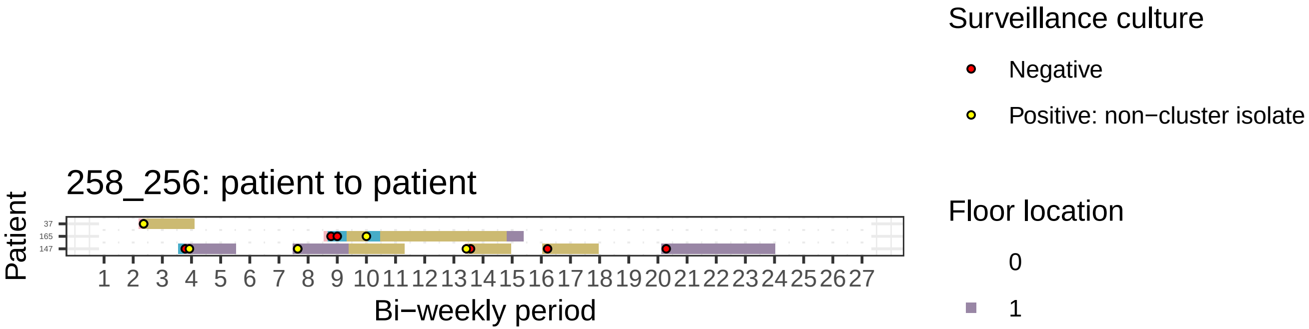
258_256: patient to patient
Patient
37
165
147
1 2 3 4 5 6 7 8 9 10 11 12 13 14 15 16 17 18 19 20 21 22 23 24 25 26 27
Bi−weekly period
Surveillance culture
Negative
Positive: non−cluster isolate
Floor location
0
1
3
4
6

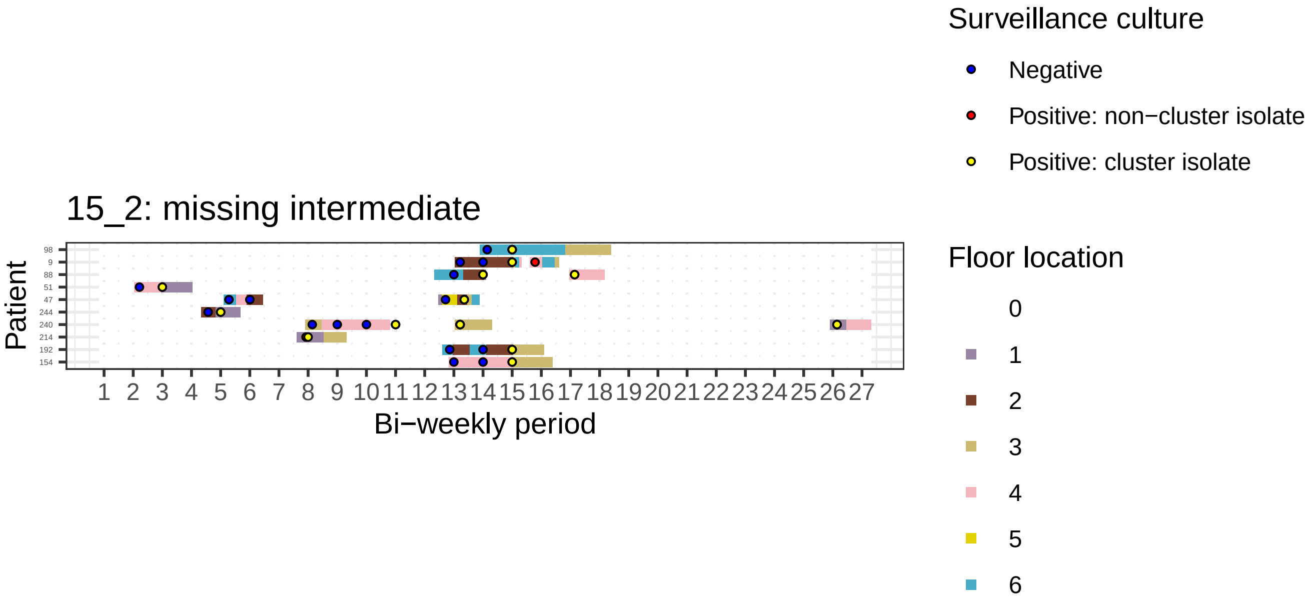

15_2: missing intermediate
Patient
98
9
88
51
47
244
240
214
192
154
1 2 3 4 5 6 7 8 9 10 11 12 13 14 15 16 17 18 19 20 21 22 23 24 25 26 27
Bi−weekly period
Surveillance culture
Negative
Positive: non−cluster isolate
Positive: cluster isolate
Floor location
0
1
2
3
4
5
6

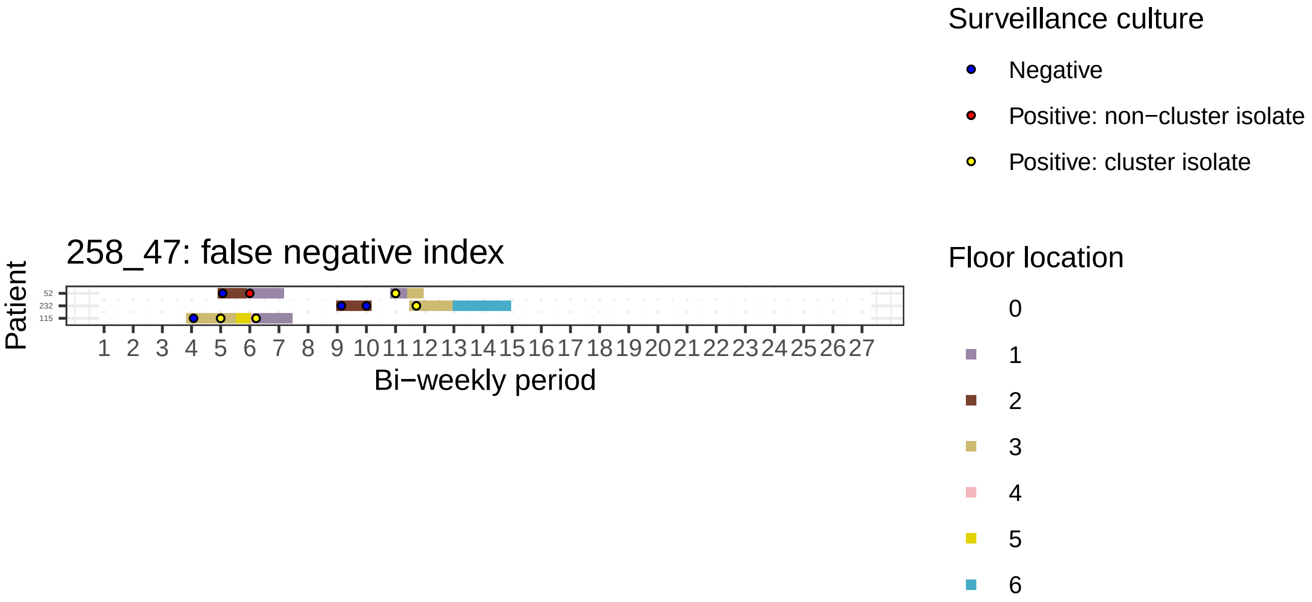

258_47: false negative index
Patient
52
232
115
1 2 3 4 5 6 7 8 9 10 11 12 13 14 15 16 17 18 19 20 21 22 23 24 25 26 27
Bi−weekly period
Surveillance culture
Negative
Positive: non−cluster isolate
Positive: cluster isolate
Floor location
0
1
2
3
4
5
6

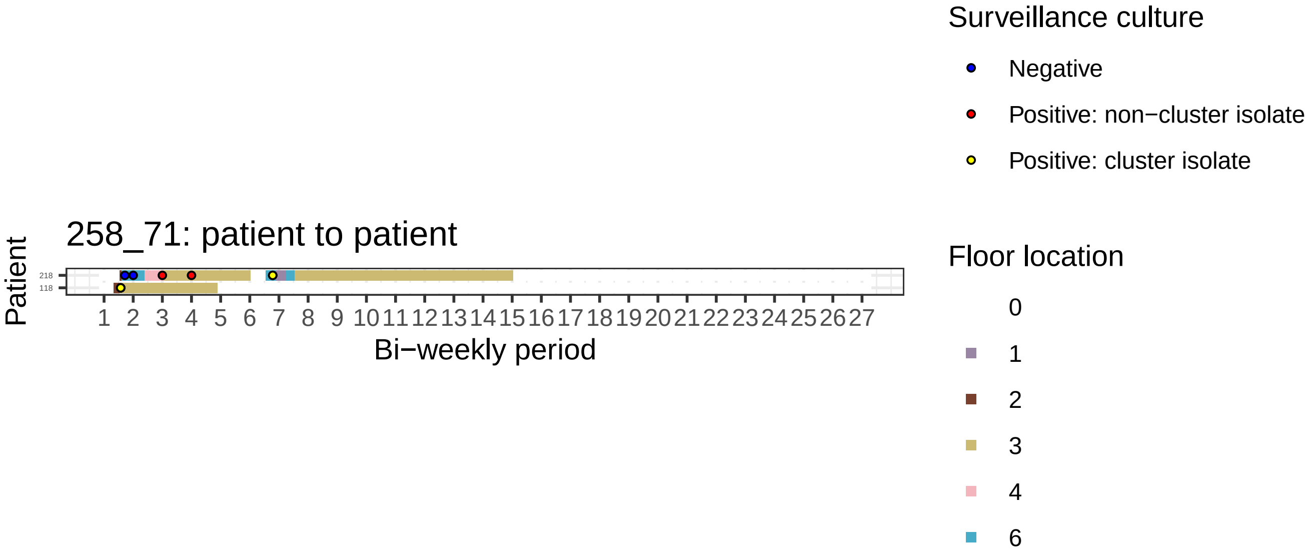

258_71: patient to patient
Patient
218
118
1 2 3 4 5 6 7 8 9 10 11 12 13 14 15 16 17 18 19 20 21 22 23 24 25 26 27
Bi−weekly period
Surveillance culture
Negative
Positive: non−cluster isolate
Positive: cluster isolate
Floor location
0
1
2
3
4
6

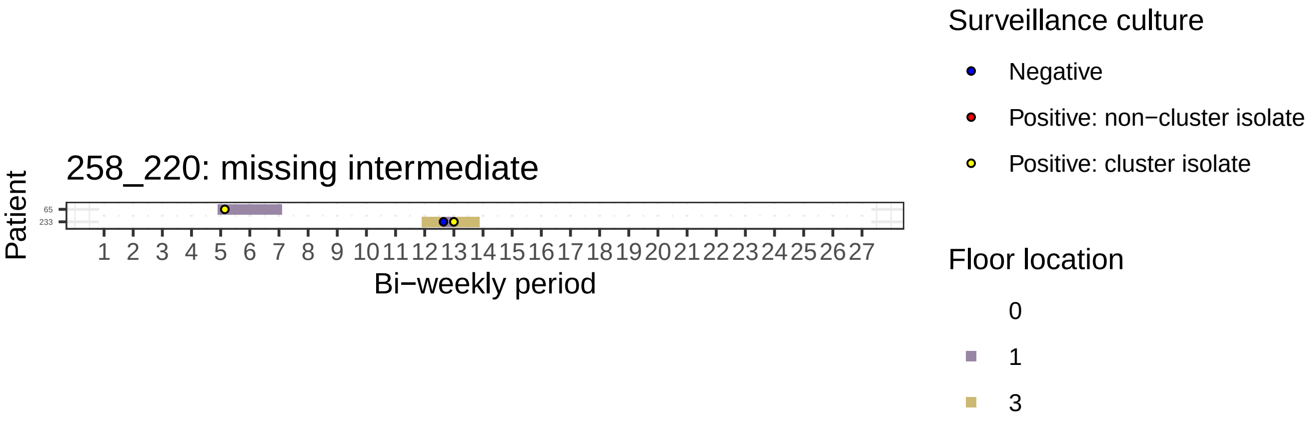

258_220: missing intermediate
Patient
65
233
1 2 3 4 5 6 7 8 9 10 11 12 13 14 15 16 17 18 19 20 21 22 23 24 25 26 27
Bi−weekly period
Surveillance culture
Negative
Positive: non−cluster isolate
Positive: cluster isolate
Floor location
0
1
3

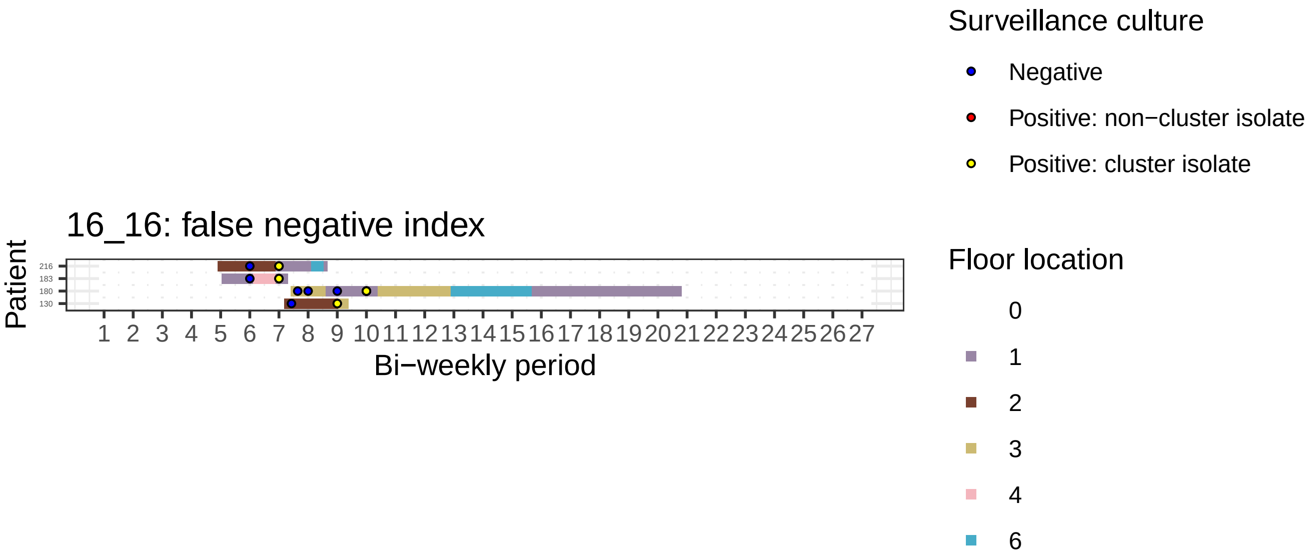

16_16: false negative index
Patient
216
183
180
130
1 2 3 4 5 6 7 8 9 10 11 12 13 14 15 16 17 18 19 20 21 22 23 24 25 26 27
Bi−weekly period
Surveillance culture
Negative
Positive: non−cluster isolate
Positive: cluster isolate
Floor location
0
1
2
3
4
6

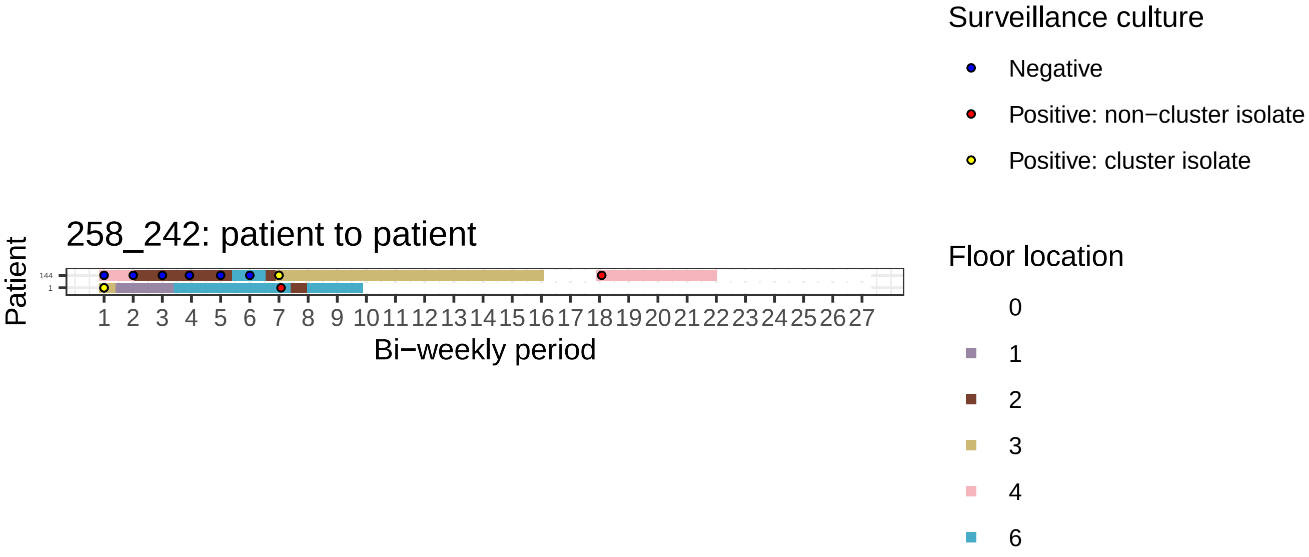
258_242: patient to patient
Patient
144
1
Bi−weekly period
1 2 3 4 5 6 7 8 9 10 11 12 13 14 15 16 17 18 19 20 21 22 23 24 25 26 27
Surveillance culture
Negative
Positive: non−cluster isolate
Positive: cluster isolate
Floor location
0
1
2
3
4
6

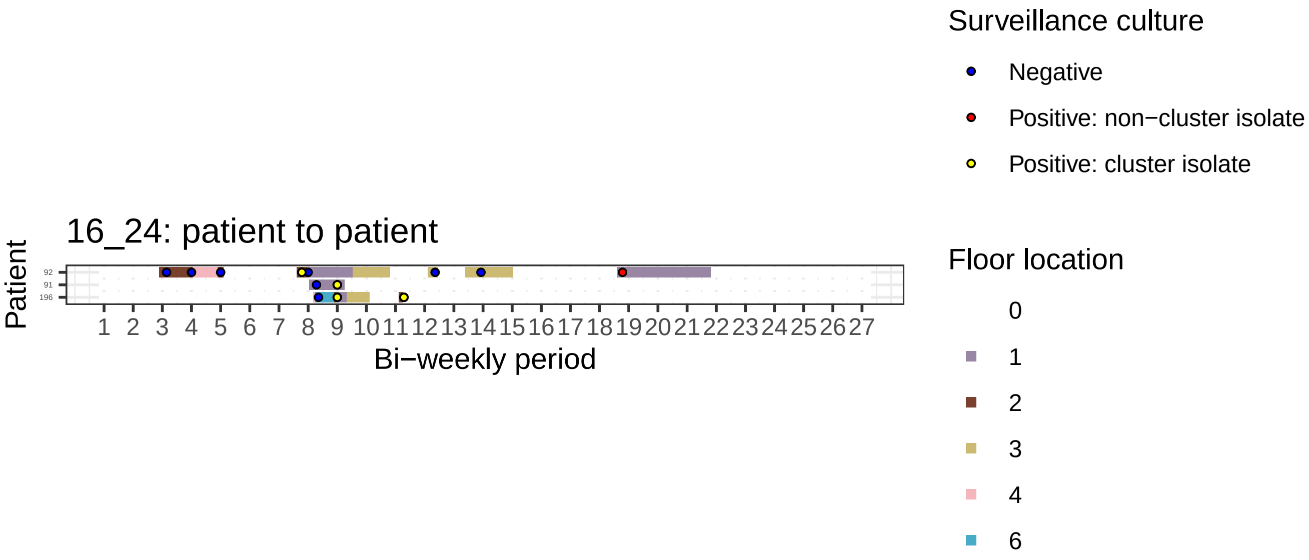
16_24: patient to patient
Patient
92
91
196
1 2 3 4 5 6 7 8 9 10 11 12 13 14 15 16 17 18 19 20 21 22 23 24 25 26 27
Bi−weekly period
Surveillance culture
Negative
Positive: non−cluster isolate
Positive: cluster isolate
Floor location
0
1
2
3
4
6

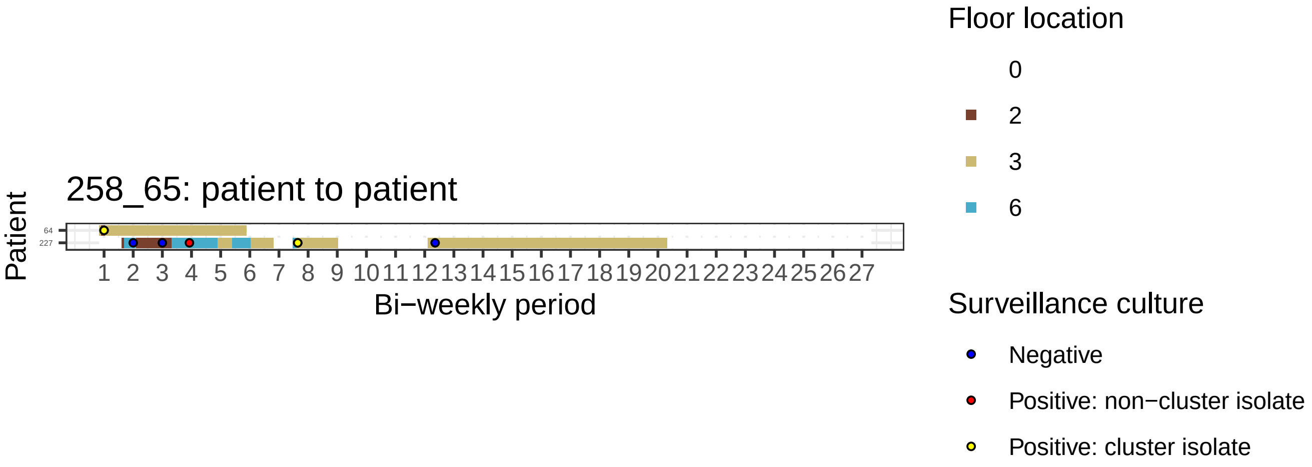

258_65: patient to patient
Patient
64
227
1 2 3 4 5 6 7 8 9 10 11 12 13 14 15 16 17 18 19 20 21 22 23 24 25 26 27
Bi−weekly period
Floor location
0
2
3
6
Surveillance culture
Negative
Positive: non−cluster isolate
Positive: cluster isolate

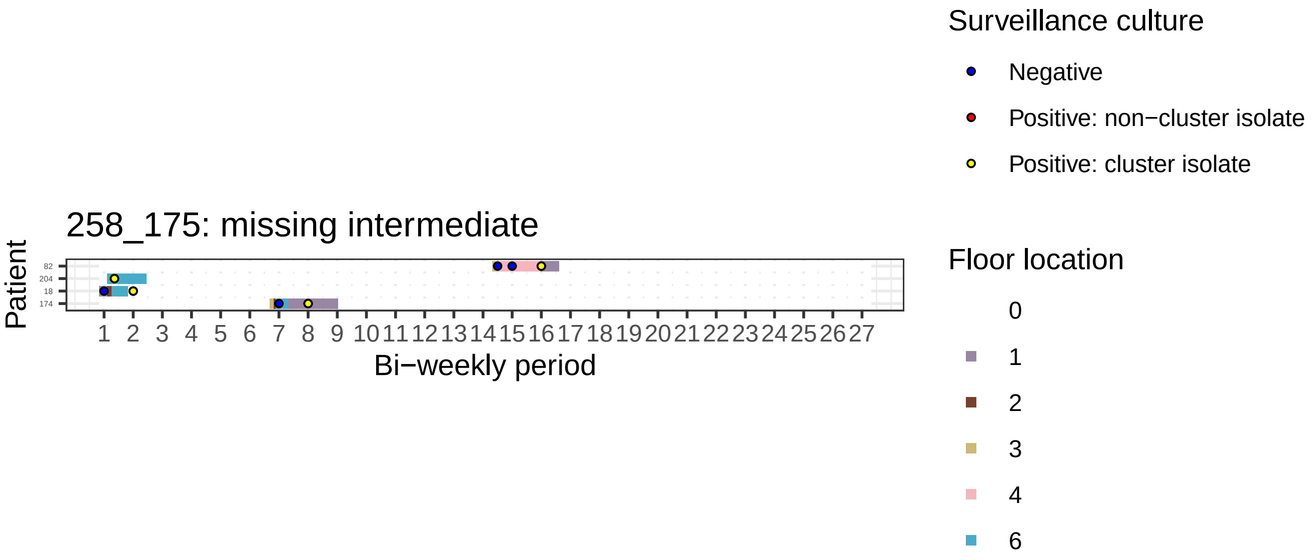

258_175: missing intermediate
Patient
82
204
18
174
1
2
3
4
5
6
7
8
9
10
11
12
13
14
15
16
17
18
19
20
21
22
23
24
25
26
27
Bi−weekly period
Surveillance culture
Negative
Positive: non−cluster isolate
Positive: cluster isolate
Floor location
0
1
2
3
4
6

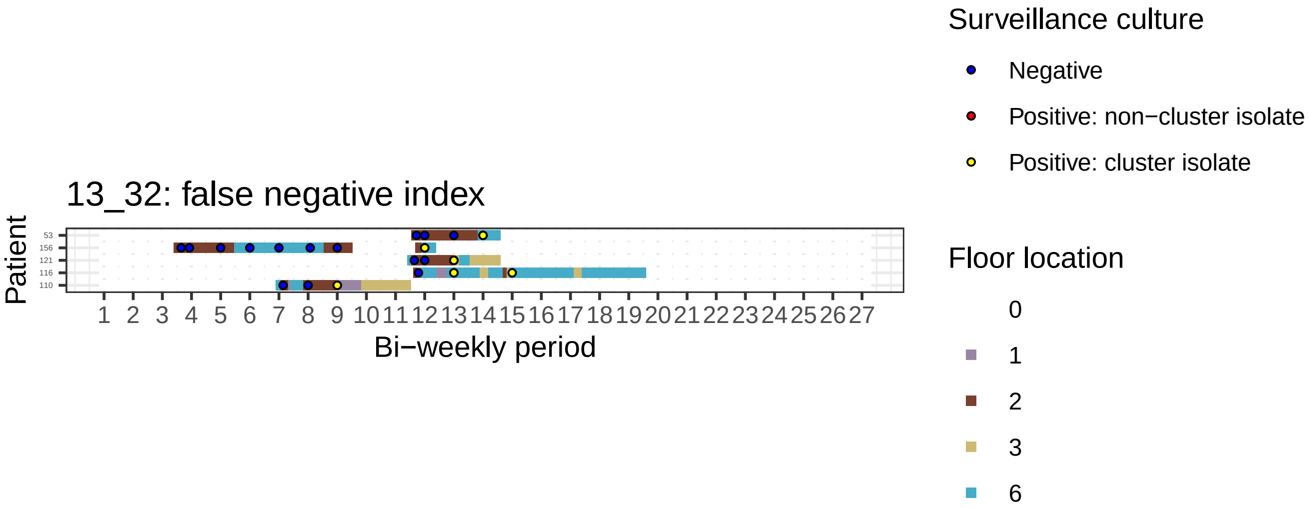
13_32: false negative index
Patient
53
156
121
116
110
1 2 3 4 5 6 7 8 9 10 11 12 13 14 15 16 17 18 19 20 21 22 23 24 25 26 27
Bi−weekly period
Surveillance culture
Negative
Positive: non−cluster isolate
Positive: cluster isolate
Floor location
0
1
2
3
6

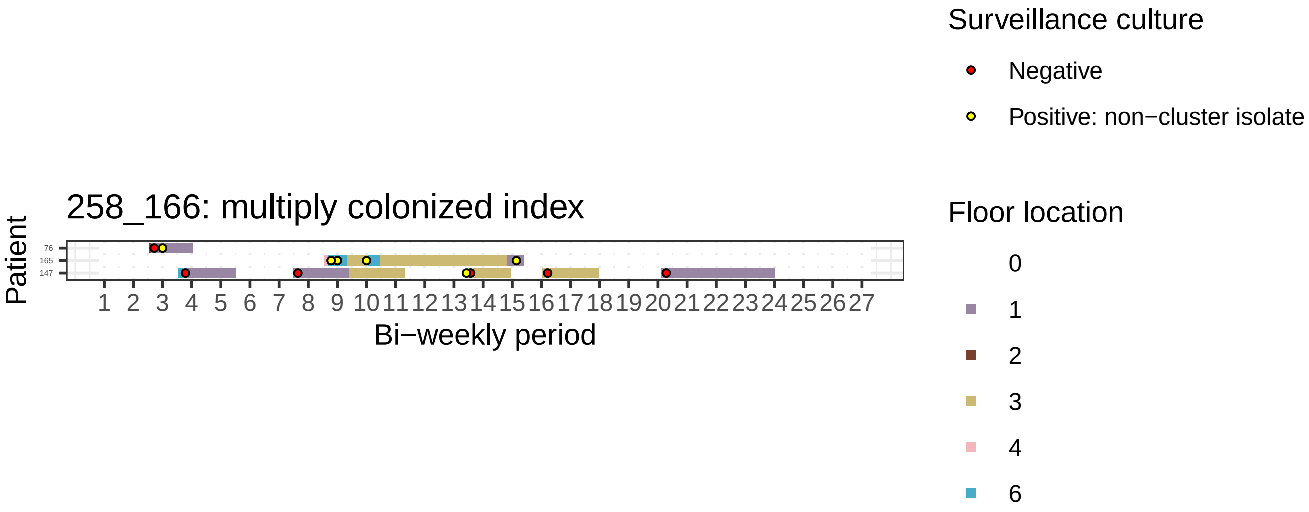
258_166: multiply colonized index
Patient
76
165
147
1 2 3 4 5 6 7 8 9 10 11 12 13 14 15 16 17 18 19 20 21 22 23 24 25 26 27
Bi−weekly period
Surveillance culture
Negative
Positive: non−cluster isolate
Floor location
0
1
2
3
4
6

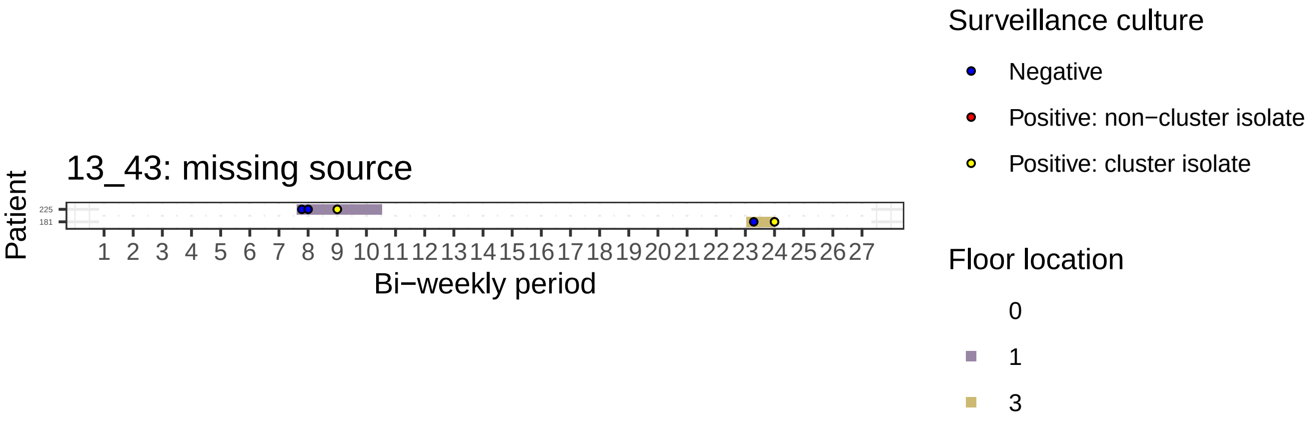

13_43: missing source
Patient
225
181
1 2 3 4 5 6 7 8 9 10 11 12 13 14 15 16 17 18 19 20 21 22 23 24 25 26 27
Bi−weekly period
Surveillance culture
Negative
Positive: non−cluster isolate
Positive: cluster isolate
Floor location
0
1
3

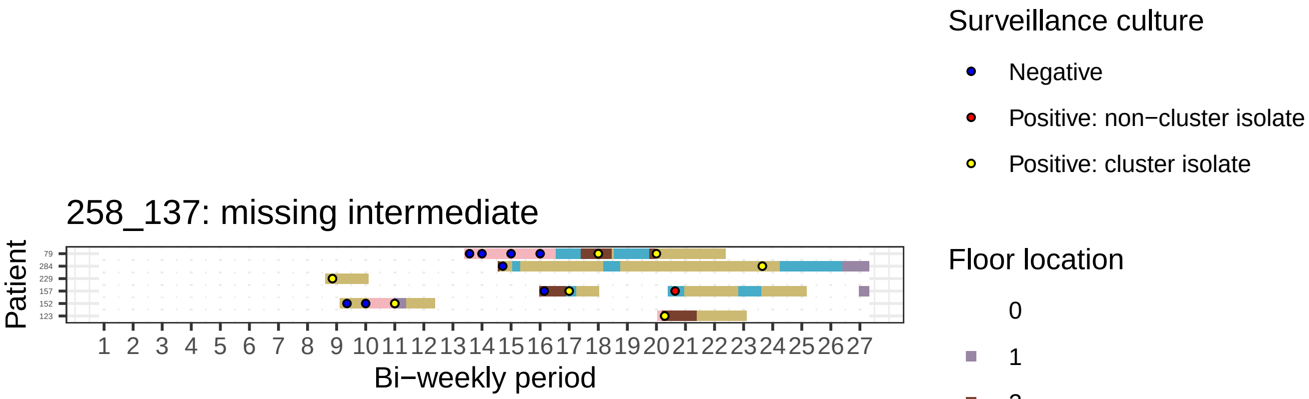
258_137: missing intermediate
Patient
79
284
229
157
152
123
1 2 3 4 5 6 7 8 9 10 11 12 13 14 15 16 17 18 19 20 21 22 23 24 25 26 27
Bi−weekly period
Surveillance culture
Negative
Positive: non−cluster isolate
Positive: cluster isolate
Floor location
0
1
2
3
4
6

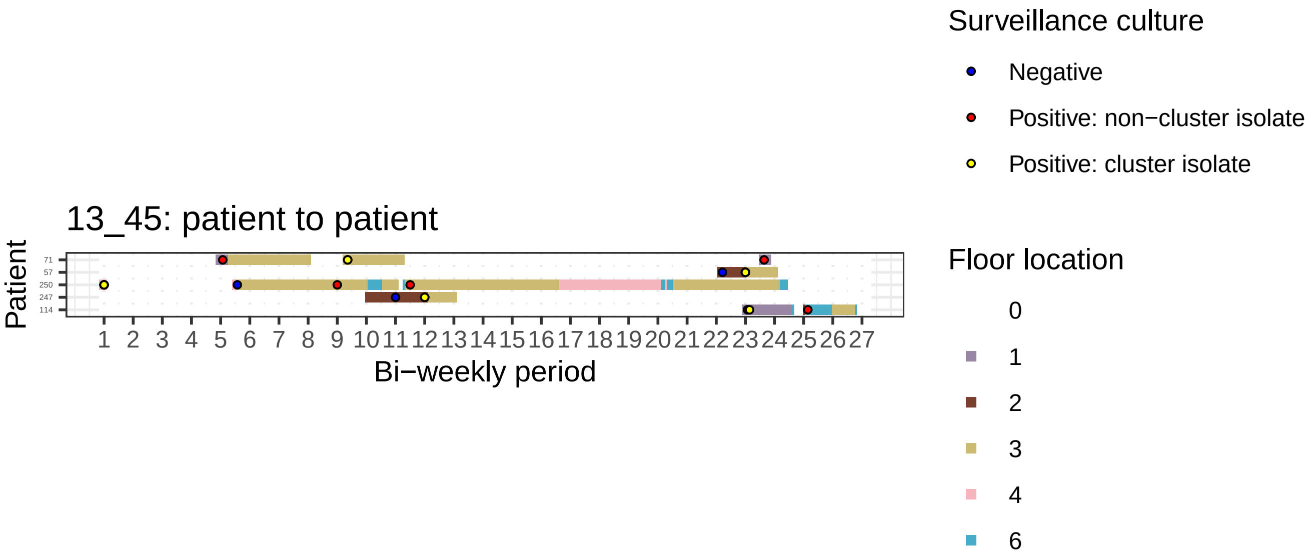

13_45: patient to patient
Patient
71
57
250
247
114
1 2 3 4 5 6 7 8 9 10 11 12 13 14 15 16 17 18 19 20 21 22 23 24 25 26 27
Bi−weekly period
Surveillance culture
Negative
Positive: non−cluster isolate
Positive: cluster isolate
Floor location
0
1
2
3
4
6

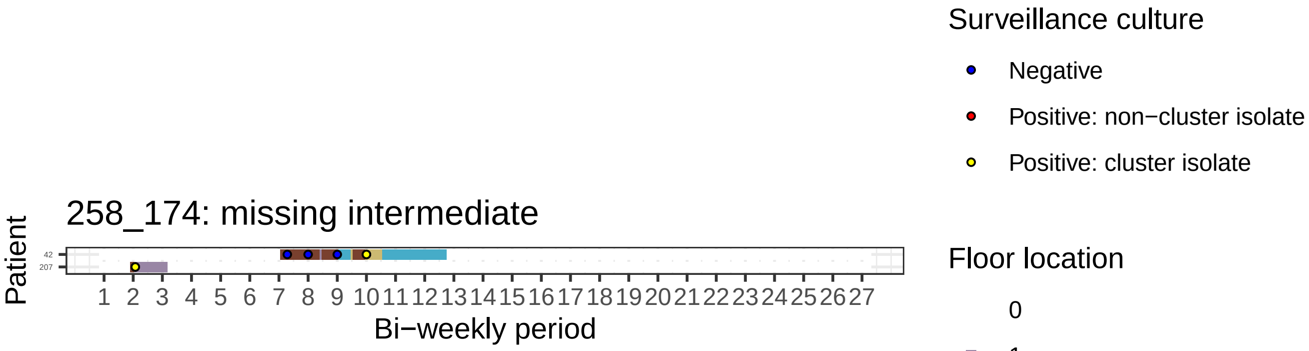

258_174: missing intermediate
Patient
42
207
1
2
3
4
5
6
7
8
9
10
11
12
13
14
15
16
17
18
19
20
21
22
23
24
25
26
27
Bi−weekly period
Surveillance culture
Negative
Positive: non−cluster isolate
Positive: cluster isolate
Floor location
0
1
2
3
6

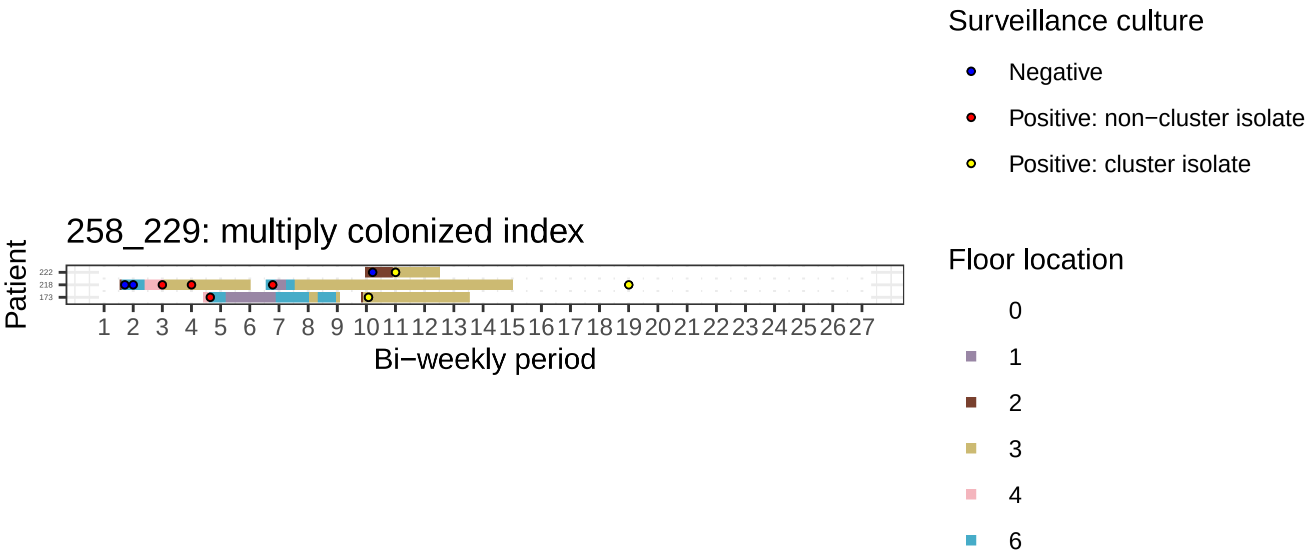
258_229: multiply colonized index
Patient
222
218
173
1
2
3
4
5
6
7
8
9
10
11
12
13
14
15
16
17
18
19
20
21
22
23
24
25
26
27
Bi−weekly period
Surveillance culture
Negative
Positive: non−cluster isolate
Positive: cluster isolate
Floor location
0
1
2
3
4
6

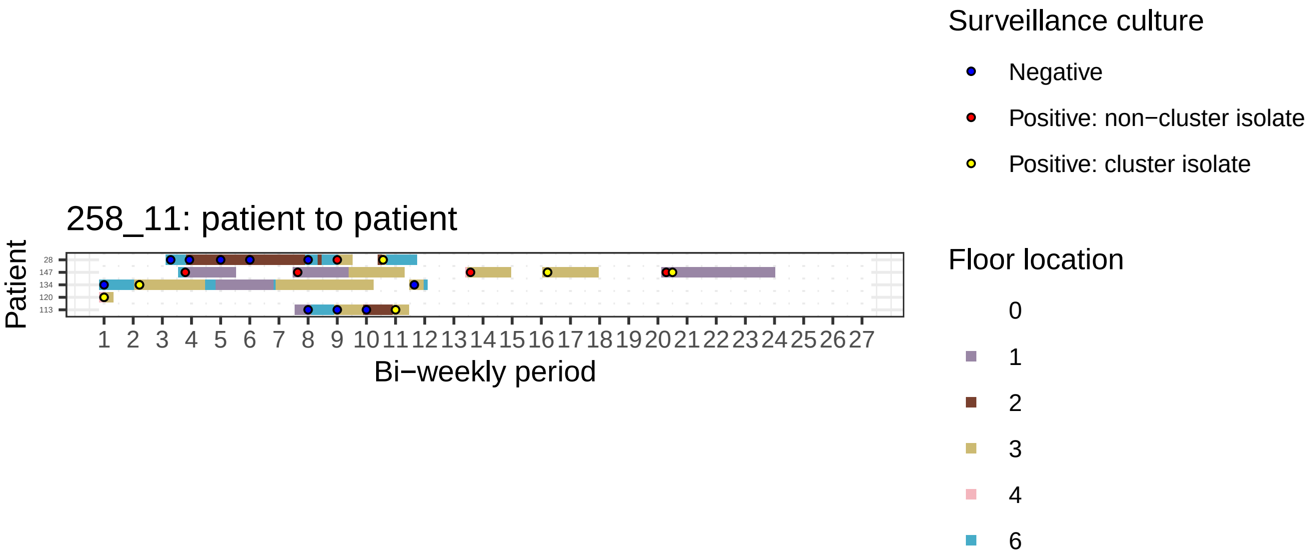

258_11: patient to patient
Patient
28
147
134
120
113
1
2
3
4
5
6
7
8
9
10
11
12
13
14
15
16
17
18
19
20
21
22
23
24
25
26
27
Bi−weekly period
Surveillance culture
Negative
Positive: non−cluster isolate
Positive: cluster isolate
Floor location
0
1
2
3
4
6

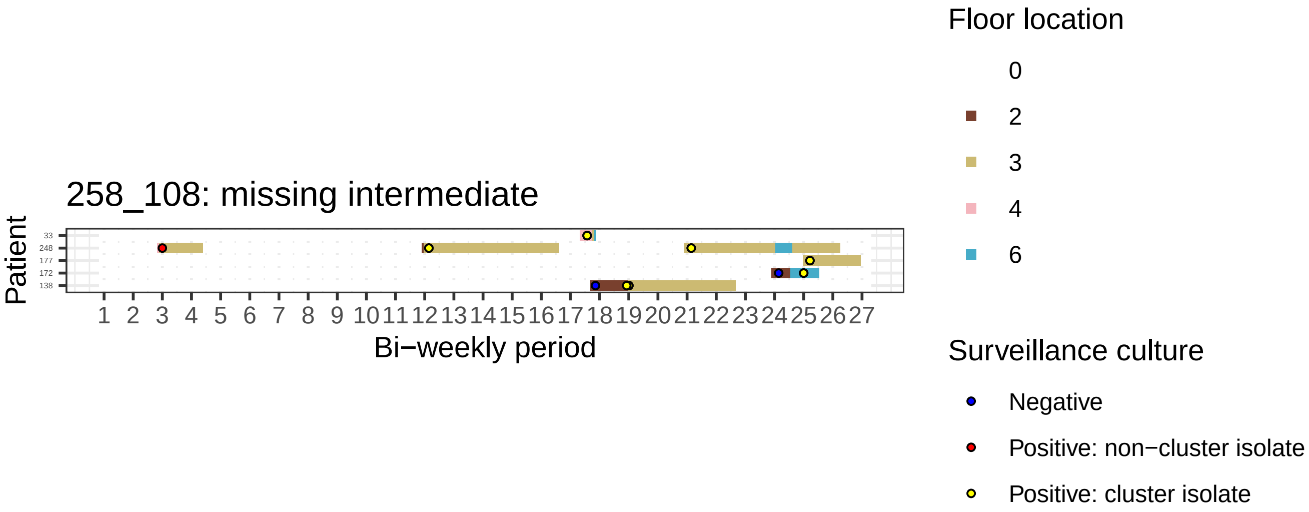
258_108: missing intermediate
Patient
33
248
177
172
138
1
2
3
4
5
6
7
8
9
10
11
12
13
14
15
16
17
18
19
20
21
22
23
24
25
26
27
Bi−weekly period
Floor location
0
2
3
4
6
Surveillance culture
Negative
Positive: non−cluster isolate
Positive: cluster isolate

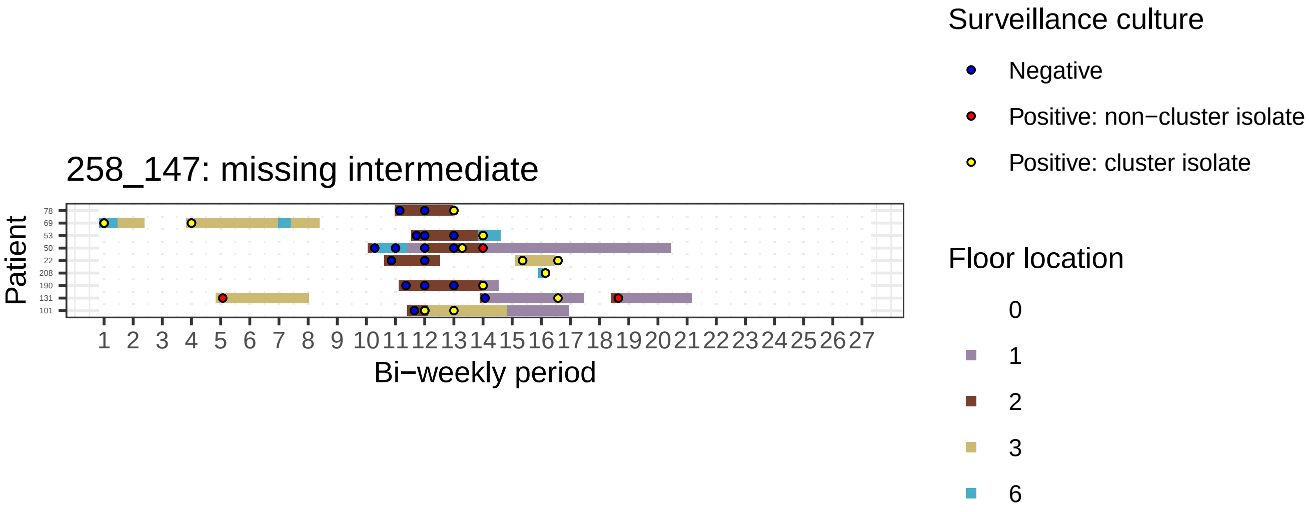
258_147: missing intermediate
Patient
78
69
53
50
22
208
190
131
101
1 2 3 4 5 6 7 8 9 10 11 12 13 14 15 16 17 18 19 20 21 22 23 24 25 26 27
Bi−weekly period
Surveillance culture
Negative
Positive: non−cluster isolate
Positive: cluster isolate
Floor location
0
1
2
3
6

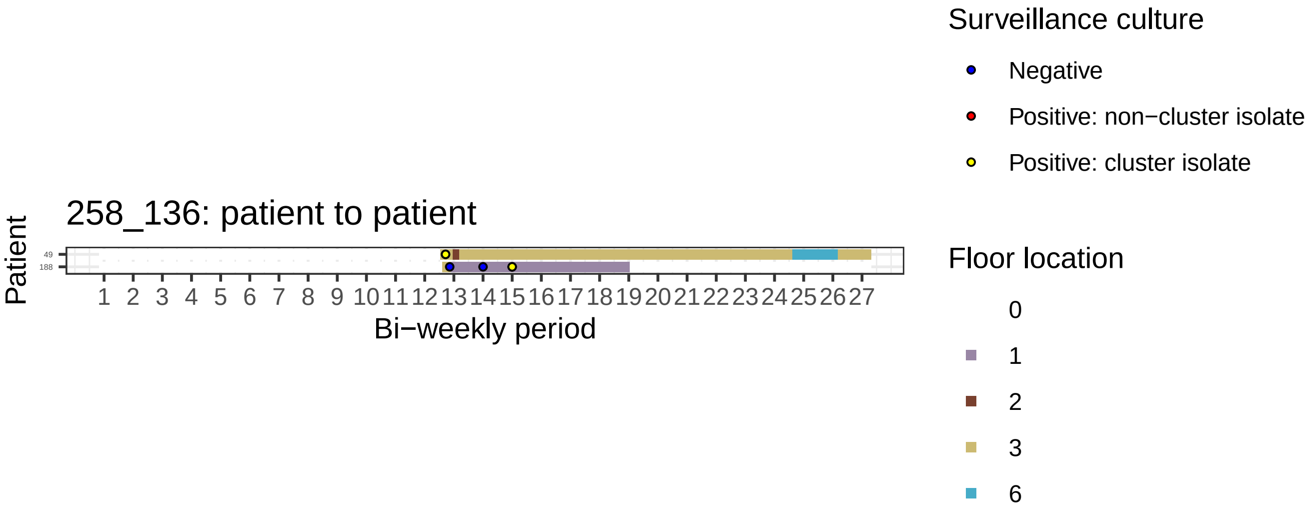

258_136: patient to patient
Patient
49
188
1 2 3 4 5 6 7 8 9 10 11 12 13 14 15 16 17 18 19 20 21 22 23 24 25 26 27
Bi−weekly period
Surveillance culture
Negative
Positive: non−cluster isolate
Positive: cluster isolate
Floor location
0
1
2
3
6

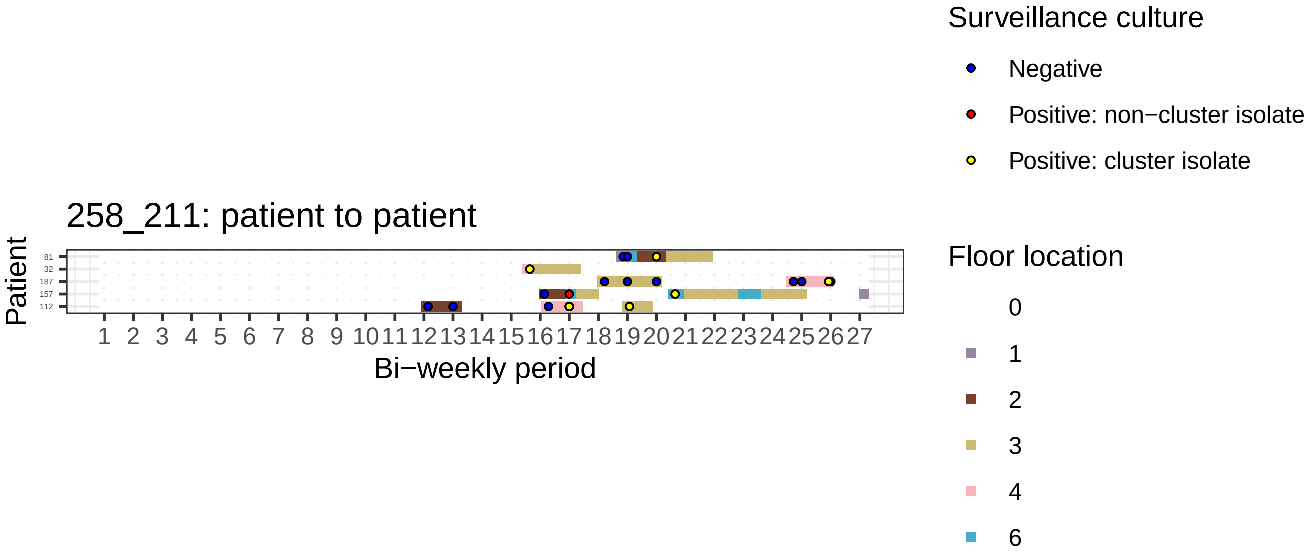
258_211: patient to patient
Patient
81
32
187
157
112
1 2 3 4 5 6 7 8 9 10 11 12 13 14 15 16 17 18 19 20 21 22 23 24 25 26 27
Bi−weekly period
Surveillance culture
Negative
Positive: non−cluster isolate
Positive: cluster isolate
Floor location
0
1
2
3
4
6

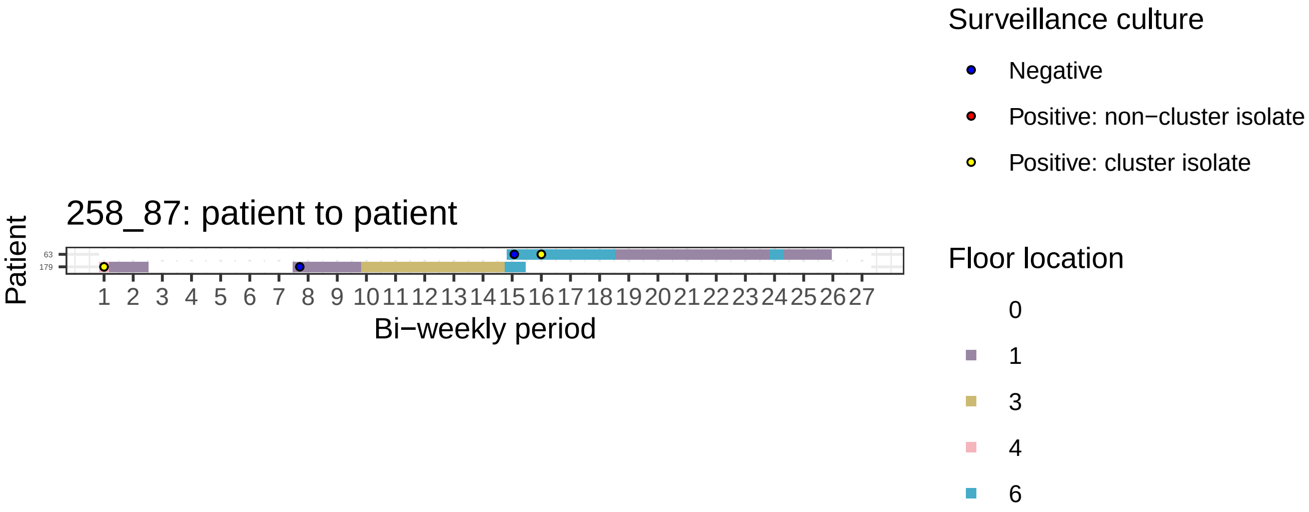

258_87: patient to patient
Patient
63
179
1 2 3 4 5 6 7 8 9 10 11 12 13 14 15 16 17 18 19 20 21 22 23 24 25 26 27
Bi−weekly period
Surveillance culture
Negative
Positive: non−cluster isolate
Positive: cluster isolate
Floor location
0
1
3
4
6

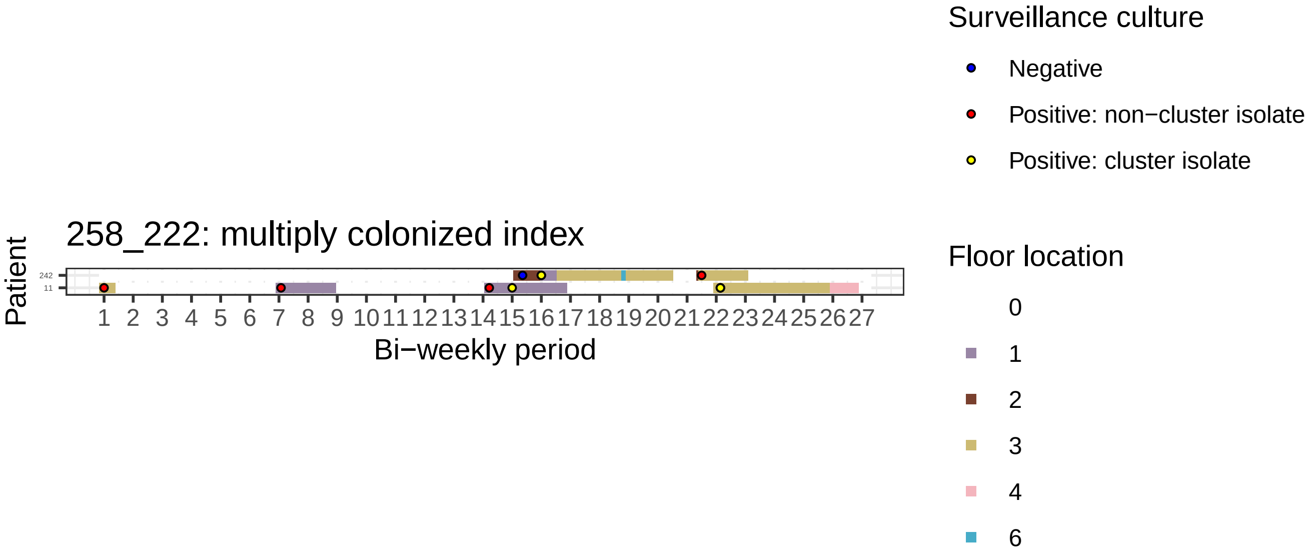

258_222: multiply colonized index
Patient
242
11
1 2 3 4 5 6 7 8 9 10 11 12 13 14 15 16 17 18 19 20 21 22 23 24 25 26 27
Bi−weekly period
Surveillance culture
Negative
Positive: non−cluster isolate
Positive: cluster isolate
Floor location
0
1
2
3
4
6

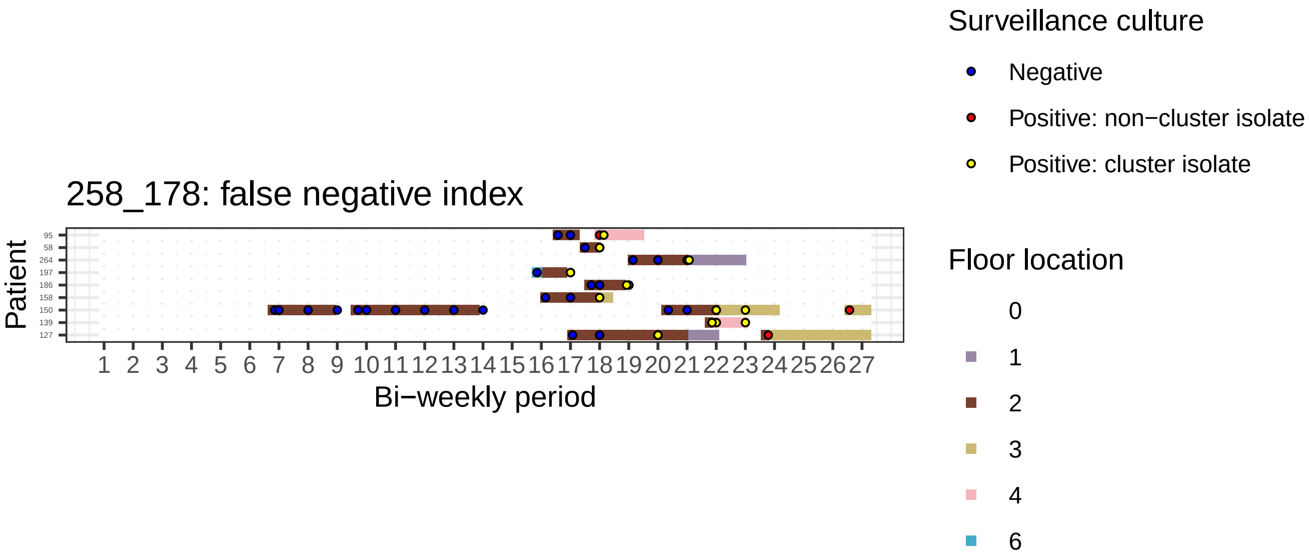

258_178: false negative index
Patient
95
58
264
197
186
158
150
139
127
1
2
3
4
5
6
7
8
9
10
11
12
13
14
15
16
17
18
19
20
21
22
23
24
25
26
27
Bi−weekly period
Surveillance culture
Negative
Positive: non−cluster isolate
Positive: cluster isolate
Floor location
0
1
2
3
4
6

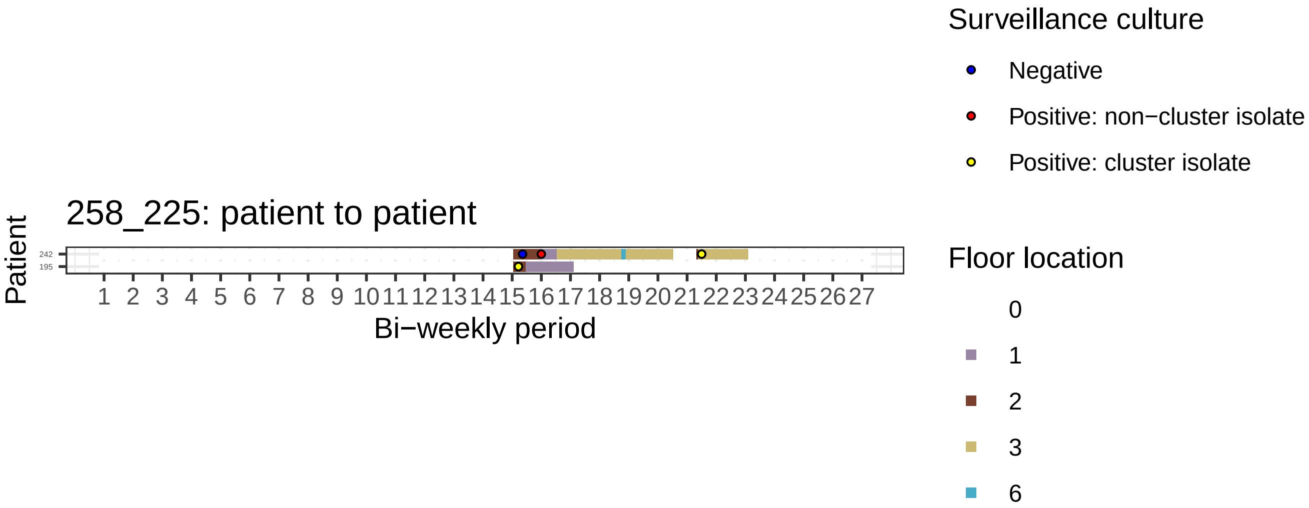

258_225: patient to patient
Patient
242
195
1
2
3
4
5
6
7
8
9
10
11
12
13
14
15
16
17
18
19
20
21
22
23
24
25
26
27
Bi−weekly period
Surveillance culture
Negative
Positive: non−cluster isolate
Positive: cluster isolate
Floor location
0
1
2
3
6

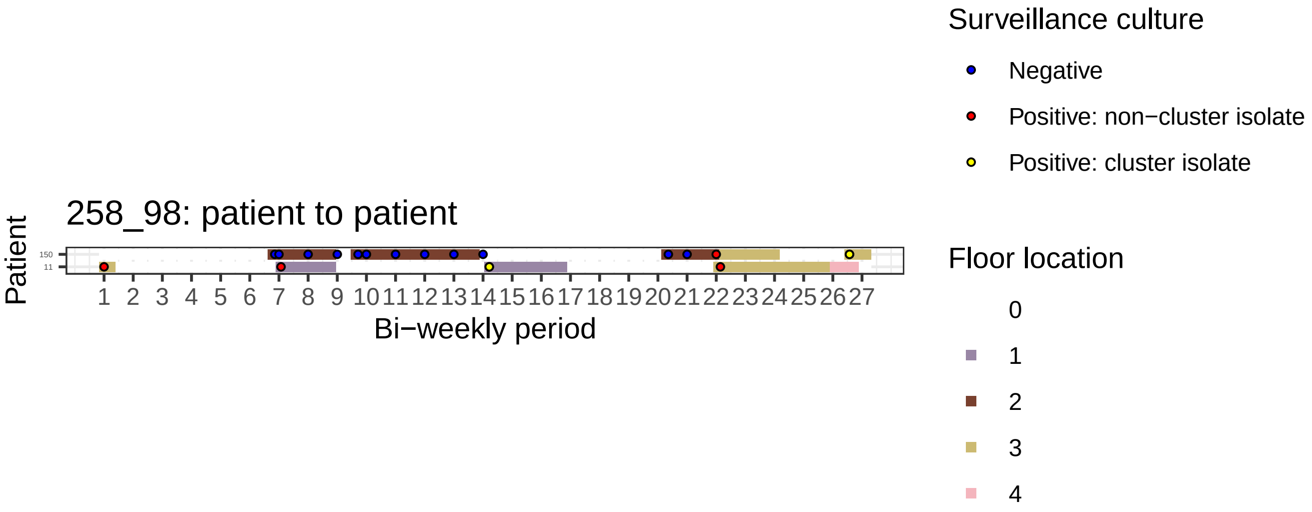

258_98: patient to patient
Patient
150
11
1
2
3
4
5
6
7
8
9
10
11
12
13
14
15
16
17
18
19
20
21
22
23
24
25
26
27
Bi−weekly period
Surveillance culture
Negative
Positive: non−cluster isolate
Positive: cluster isolate
Floor location
0
1
2
3
4

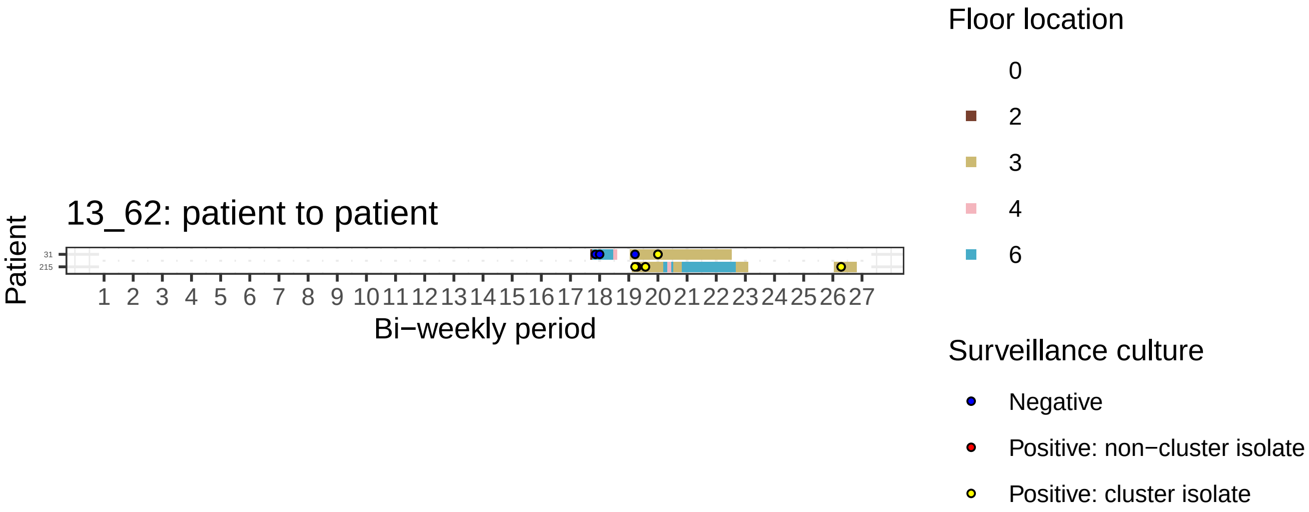

13_62: patient to patient
Patient
31
215
1 2 3 4 5 6 7 8 9 10 11 12 13 14 15 16 17 18 19 20 21 22 23 24 25 26 27
Bi−weekly period
Floor location
0
2
3
4
6
Surveillance culture
Negative
Positive: non−cluster isolate
Positive: cluster isolate

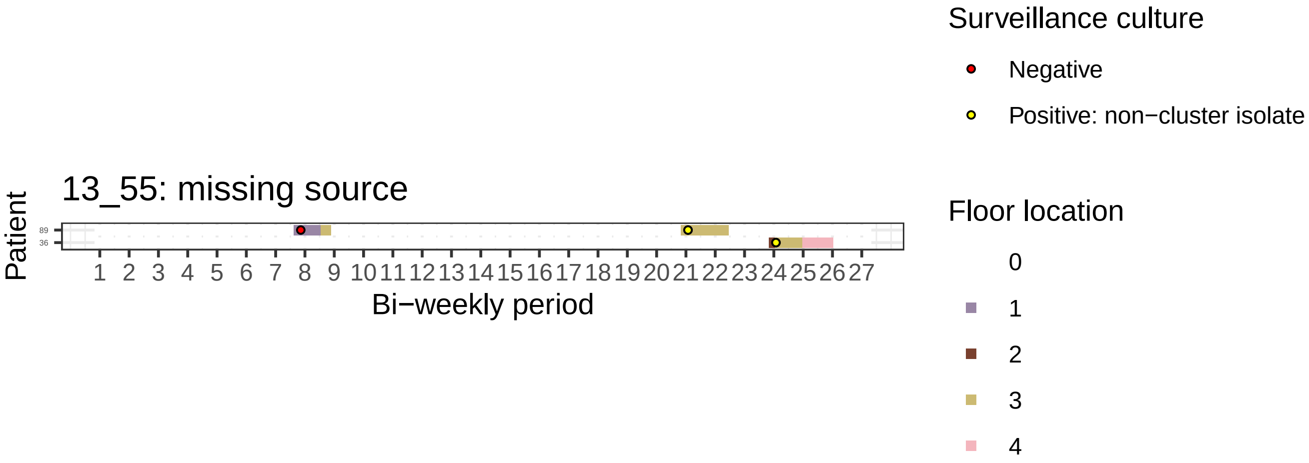

13_55: missing source
Patient
89
36
1 2 3 4 5 6 7 8 9 10 11 12 13 14 15 16 17 18 19 20 21 22 23 24 25 26 27
Bi−weekly period
Surveillance culture
Negative
Positive: non−cluster isolate
Floor location
0
1
2
3
4

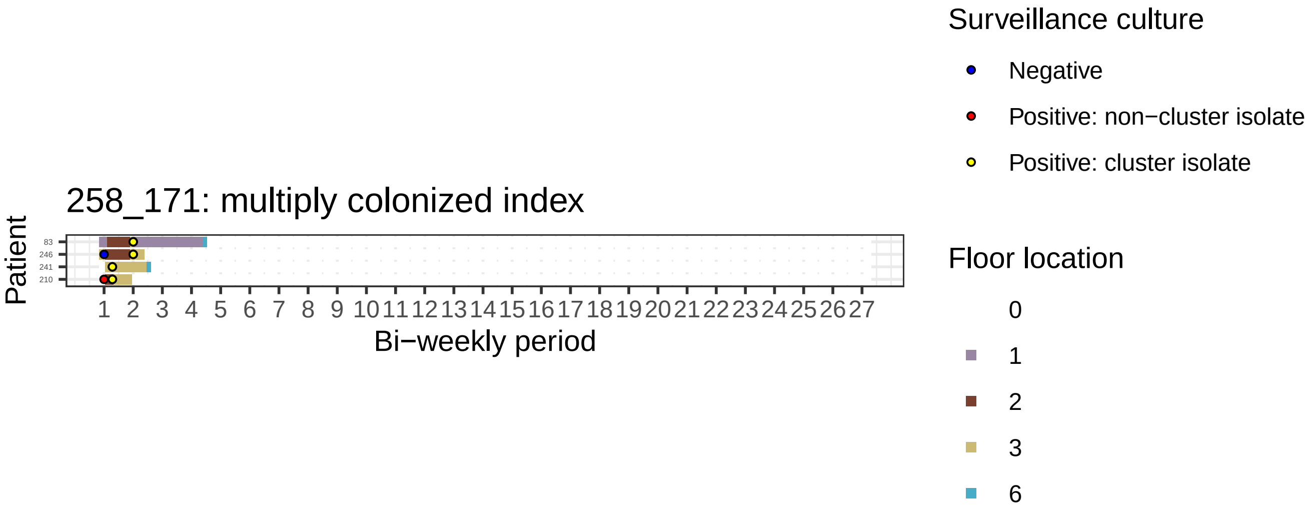

258_171: multiply colonized index
Patient
83
246
241
210
1 2 3 4 5 6 7 8 9 10 11 12 13 14 15 16 17 18 19 20 21 22 23 24 25 26 27
Bi−weekly period
Surveillance culture
Negative
Positive: non−cluster isolate
Positive: cluster isolate
Floor location
0
1
2
3
6

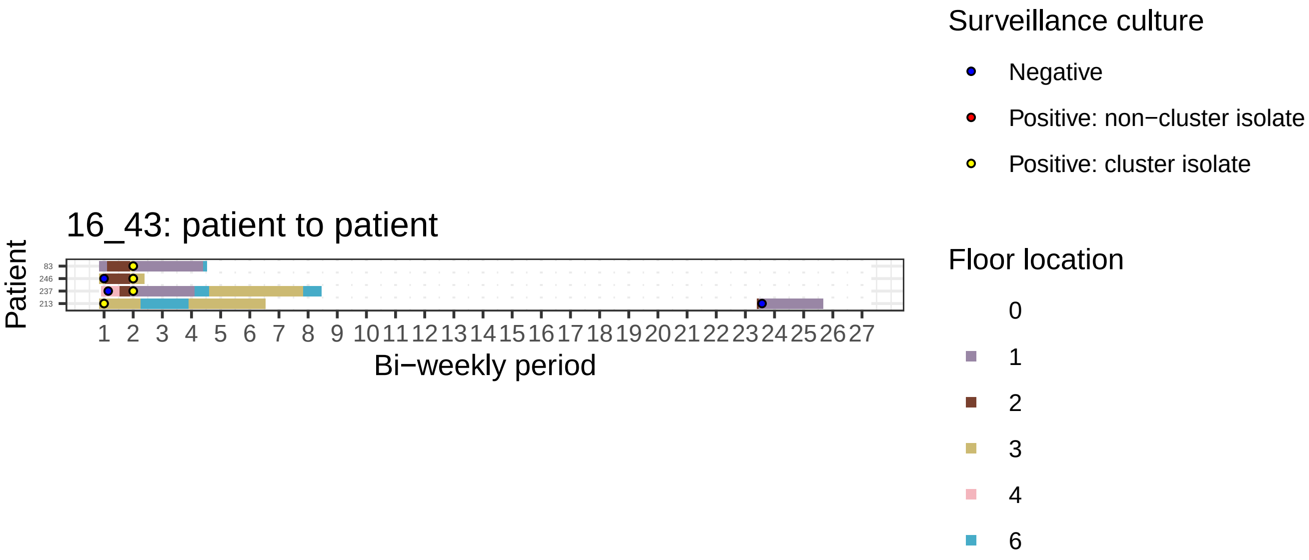

16_43: patient to patient
Patient
83
246
237
213
1 2 3 4 5 6 7 8 9 10 11 12 13 14 15 16 17 18 19 20 21 22 23 24 25 26 27
Bi−weekly period
Surveillance culture
Negative
Positive: non−cluster isolate
Positive: cluster isolate
Floor location
0
1
2
3
4
6

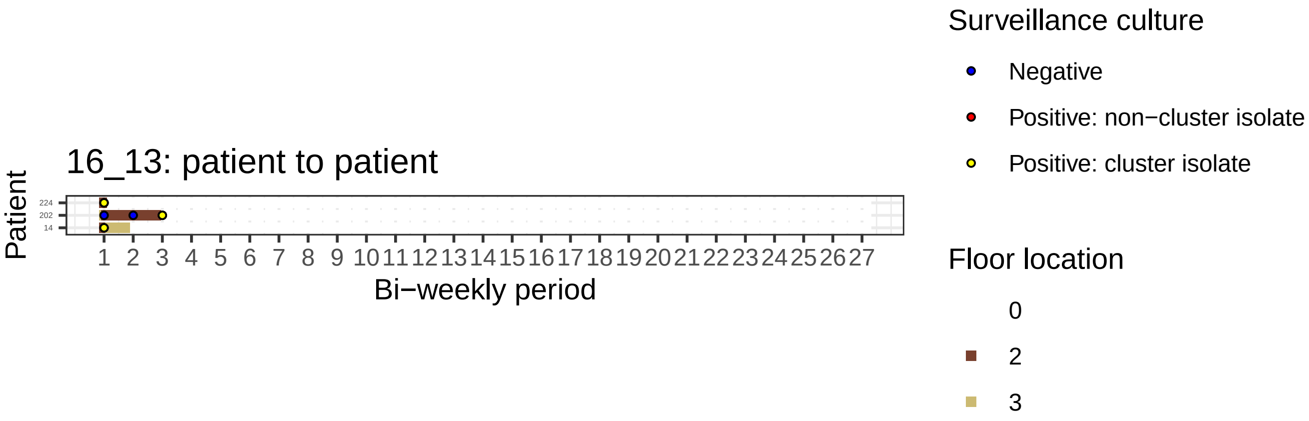
16_13: patient to patient
Patient
224
202
14
1 2 3 4 5 6 7 8 9 10 11 12 13 14 15 16 17 18 19 20 21 22 23 24 25 26 27
Bi−weekly period
Surveillance culture
Negative
Positive: non−cluster isolate
Positive: cluster isolate
Floor location
0
2
3

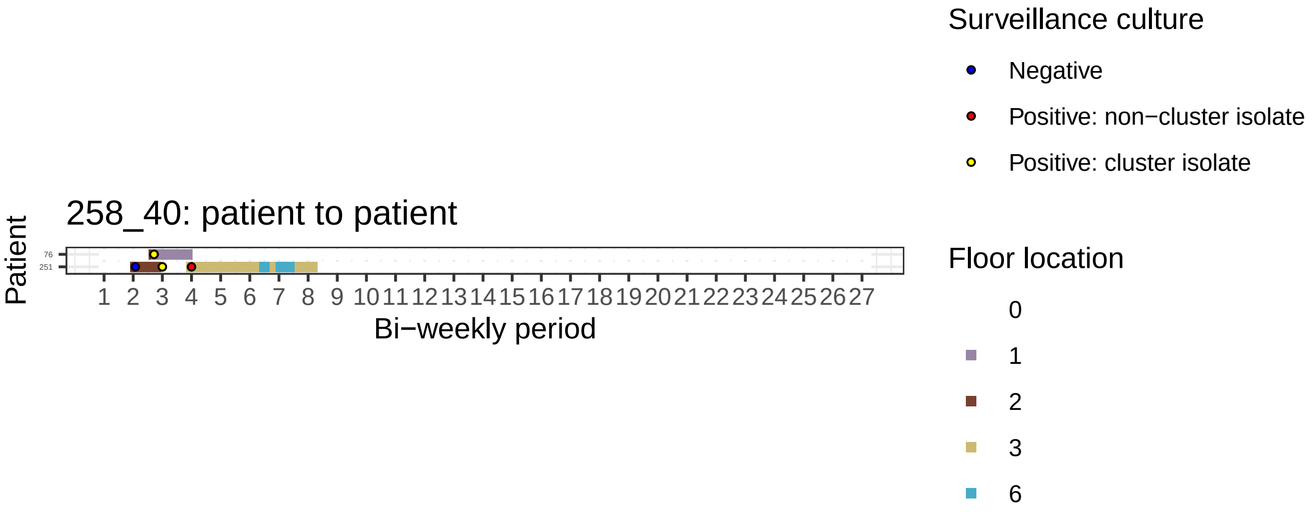

258_40: patient to patient
Patient
76
251
1
2
3
4
5
6
7
8
9
10
11
12
13
14
15
16
17
18
19
20
21
22
23
24
25
26
27
Bi−weekly period
Surveillance culture
Negative
Positive: non−cluster isolate
Positive: cluster isolate
Floor location
0
1
2
3
6

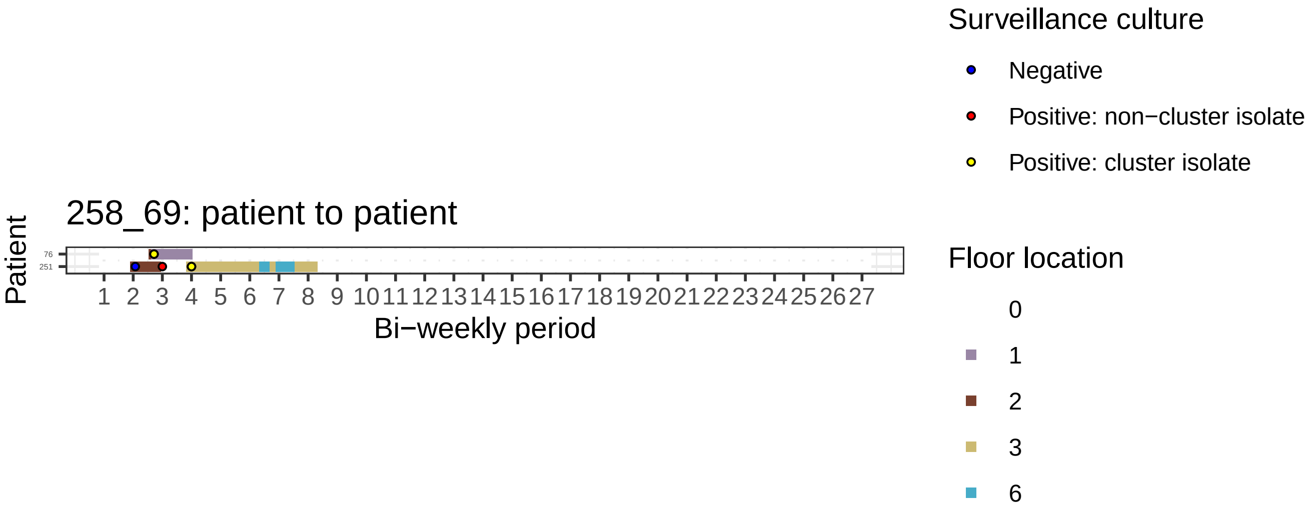

258_69: patient to patient
Patient
76
251
1
2
3
4
5
6
7
8
9
10
11
12
13
14
15
16
17
18
19
20
21
22
23
24
25
26
27
Bi−weekly period
Surveillance culture
Negative
Positive: non−cluster isolate
Positive: cluster isolate
Floor location
0
1
2
3
6

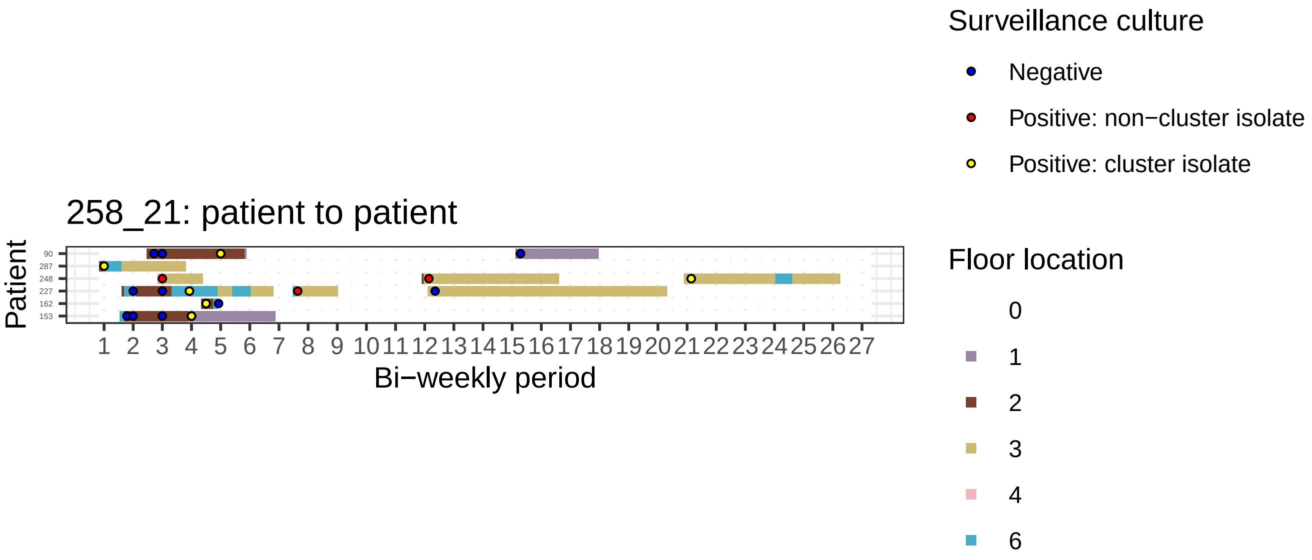

258_21: patient to patient
Patient
90
287
248
227
162
153
1 2 3 4 5 6 7 8 9 10 11 12 13 14 15 16 17 18 19 20 21 22 23 24 25 26 27
Bi−weekly period
Surveillance culture
Negative
Positive: non−cluster isolate
Positive: cluster isolate
Floor location
0
1
2
3
4
6

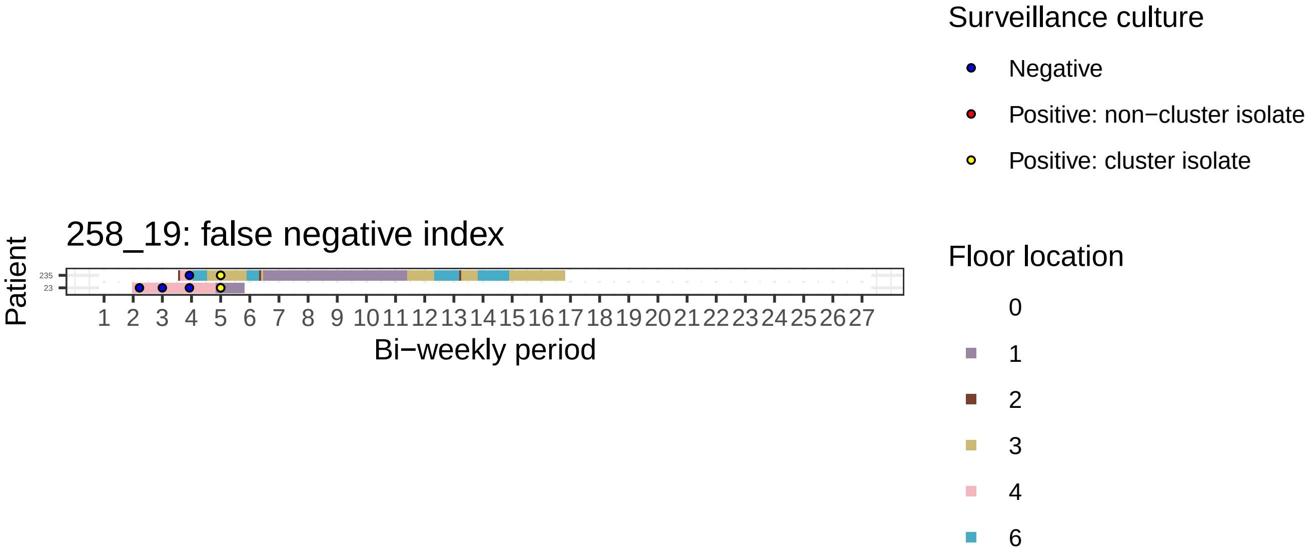

258_19: false negative index
Patient
235
23
1 2 3 4 5 6 7 8 9 10 11 12 13 14 15 16 17 18 19 20 21 22 23 24 25 26 27
Bi−weekly period
Surveillance culture
Negative
Positive: non−cluster isolate
Positive: cluster isolate
Floor location
0
1
2
3
4
6

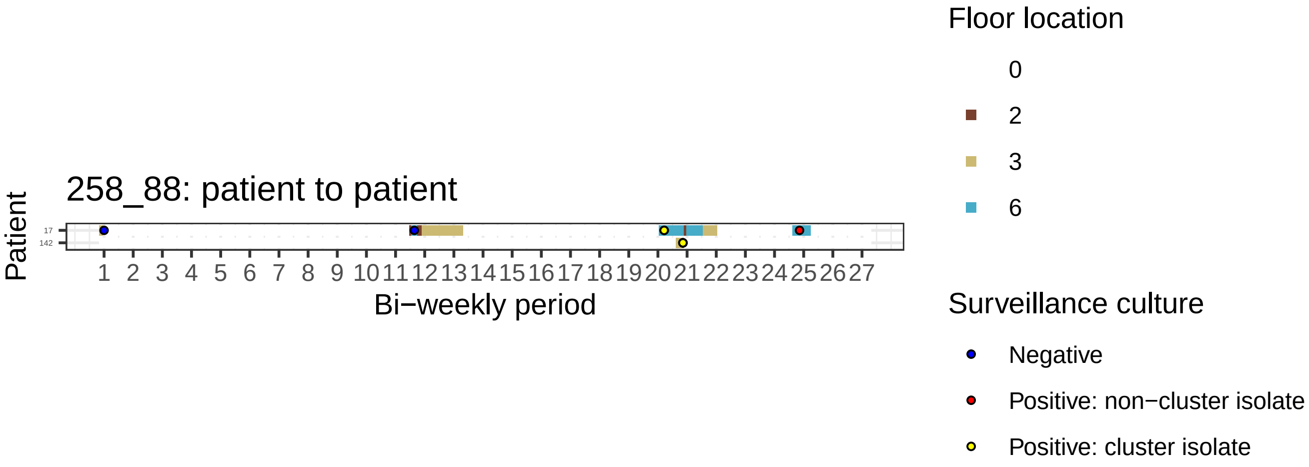

258_88: patient to patient
Patient
17
142
1 2 3 4 5 6 7 8 9 10 11 12 13 14 15 16 17 18 19 20 21 22 23 24 25 26 27
Bi−weekly period
Floor location
0
2
3
6
Surveillance culture
Negative
Positive: non−cluster isolate
Positive: cluster isolate

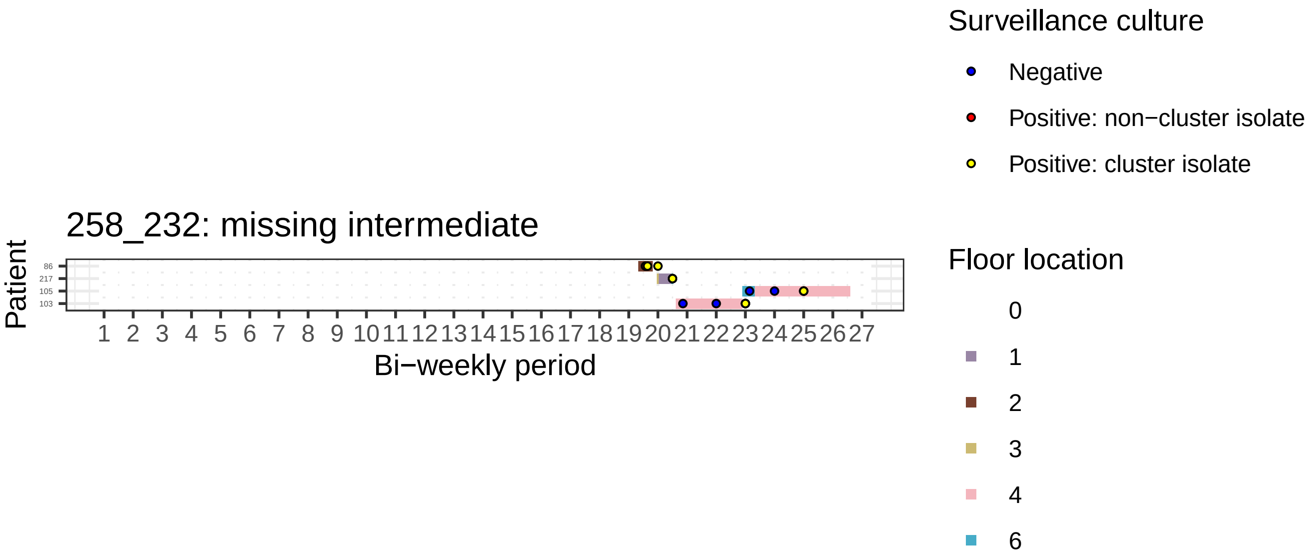

258_232: missing intermediate
Patient
86
217
105
103
1 2 3 4 5 6 7 8 9 10 11 12 13 14 15 16 17 18 19 20 21 22 23 24 25 26 27
Bi−weekly period
Surveillance culture
Negative
Positive: non−cluster isolate
Positive: cluster isolate
Floor location
0
1
2
3
4
6

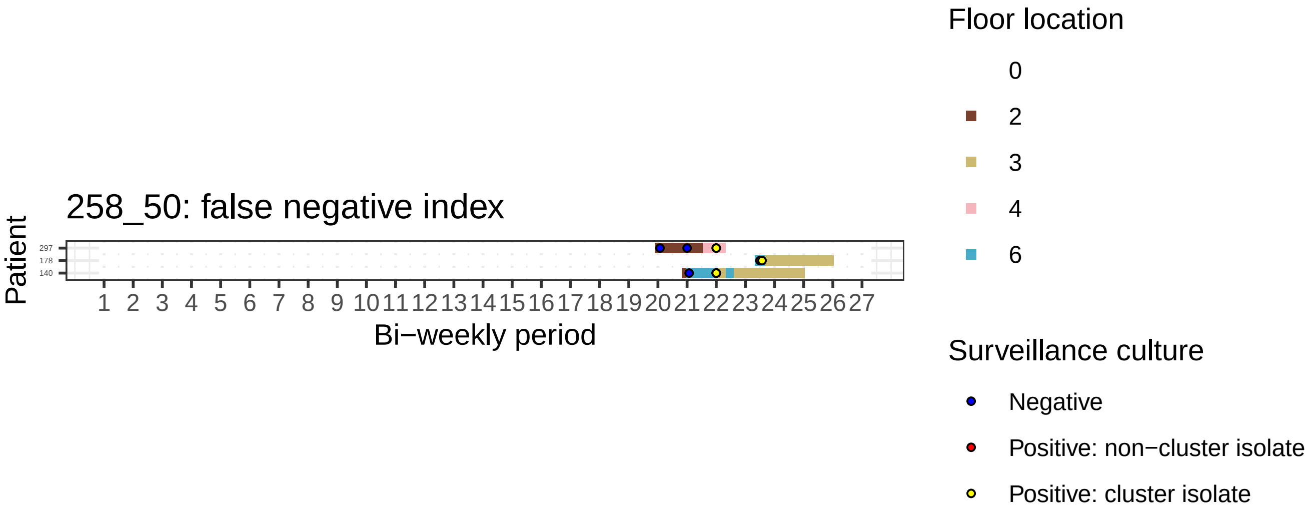

258_50: false negative index
Patient
297
178
140
1 2 3 4 5 6 7 8 9 10 11 12 13 14 15 16 17 18 19 20 21 22 23 24 25 26 27
Bi−weekly period
Floor location
0
2
3
4
6
Surveillance culture
Negative
Positive: non−cluster isolate
Positive: cluster isolate

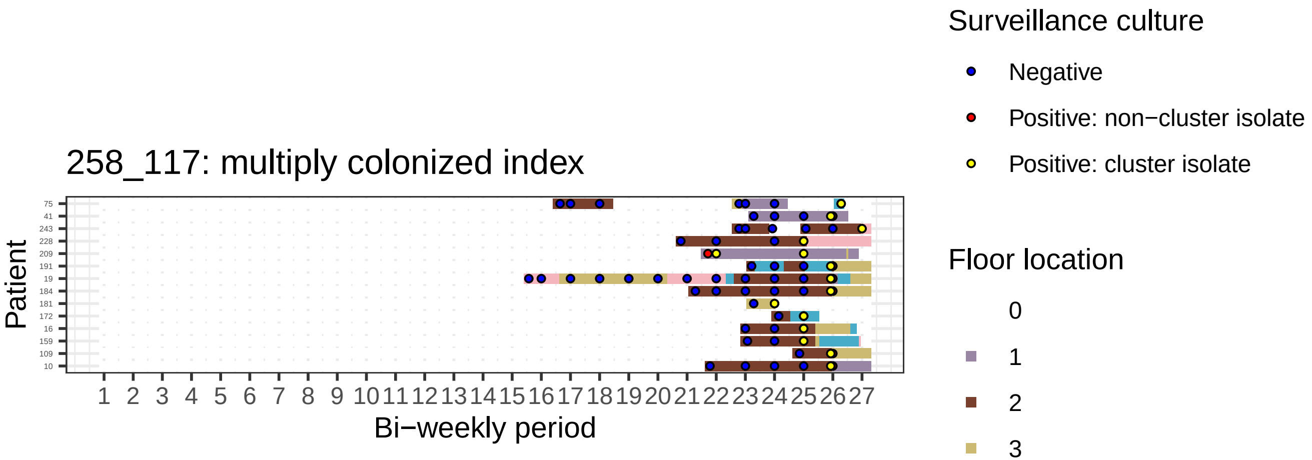
258_117: multiply colonized index
Patient
75
41
243
228
209
191
19
184
181
172
16
159
109
10
1 2 3 4 5 6 7 8 9 10 11 12 13 14 15 16 17 18 19 20 21 22 23 24 25 26 27
Bi−weekly period
Surveillance culture
Negative
Positive: non−cluster isolate
Positive: cluster isolate
Floor location
0
1
2
3
4
6

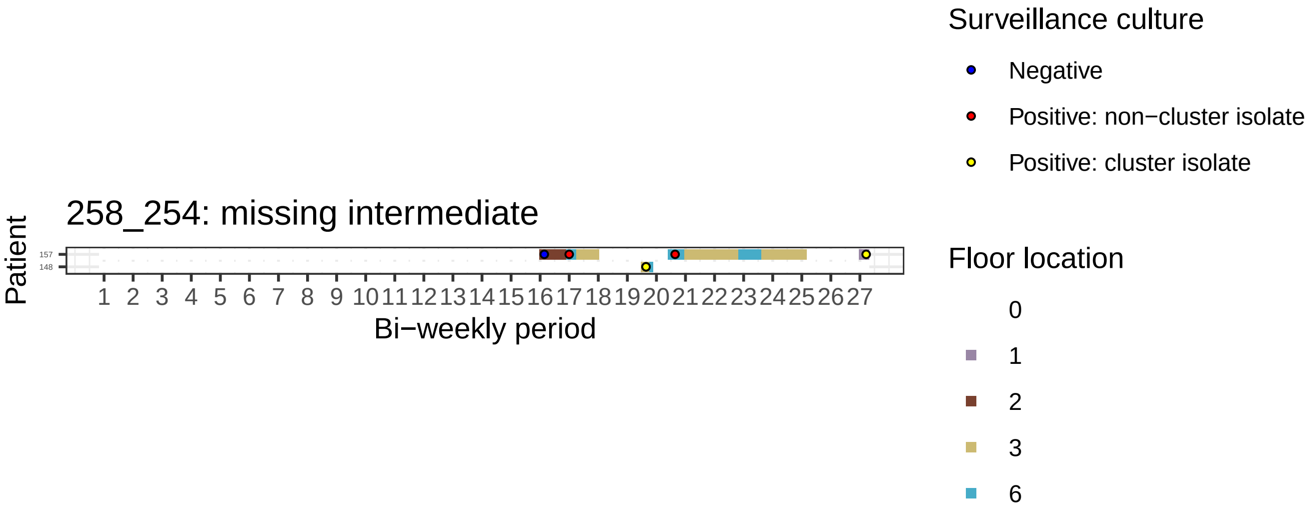

258_254: missing intermediate
Patient
157
148
1 2 3 4 5 6 7 8 9 10 11 12 13 14 15 16 17 18 19 20 21 22 23 24 25 26 27
Bi−weekly period
Surveillance culture
Negative
Positive: non−cluster isolate
Positive: cluster isolate
Floor location
0
1
2
3
6

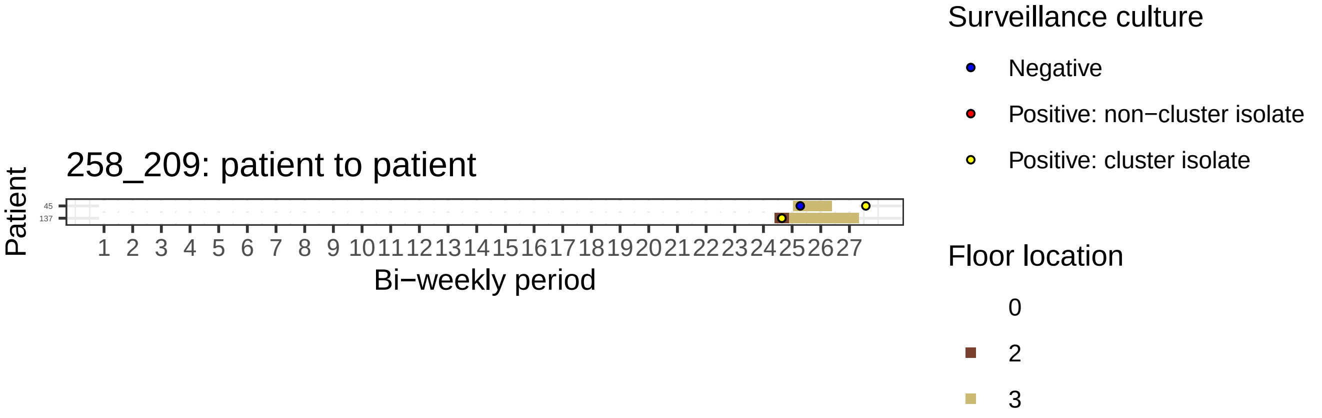

258_209: patient to patient
Patient
45
137
1
2
3
4
5
6
7
8
9
10
11
12
13
14
15
16
17
18
19
20
21
22
23
24
25
26
27
Bi−weekly period
Surveillance culture
Negative
Positive: non−cluster isolate
Positive: cluster isolate
Floor location
0
2
3

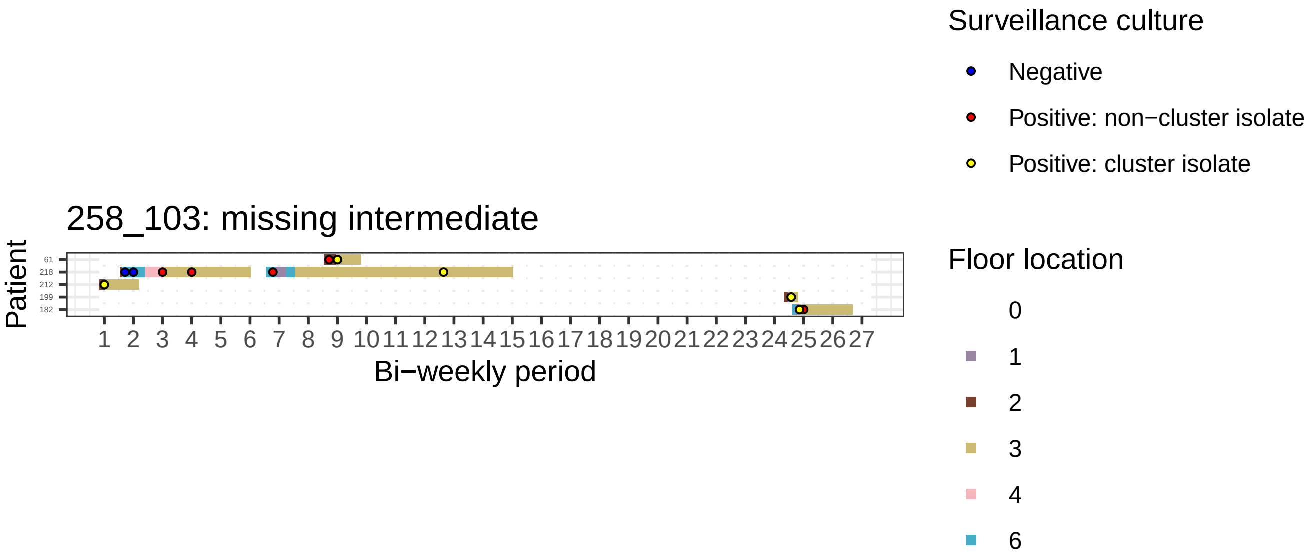
258_103: missing intermediate
Patient
61
218
212
199
182
1 2 3 4 5 6 7 8 9 10 11 12 13 14 15 16 17 18 19 20 21 22 23 24 25 26 27
Bi−weekly period
Surveillance culture
Negative
Positive: non−cluster isolate
Positive: cluster isolate
Floor location
0
1
2
3
4
6

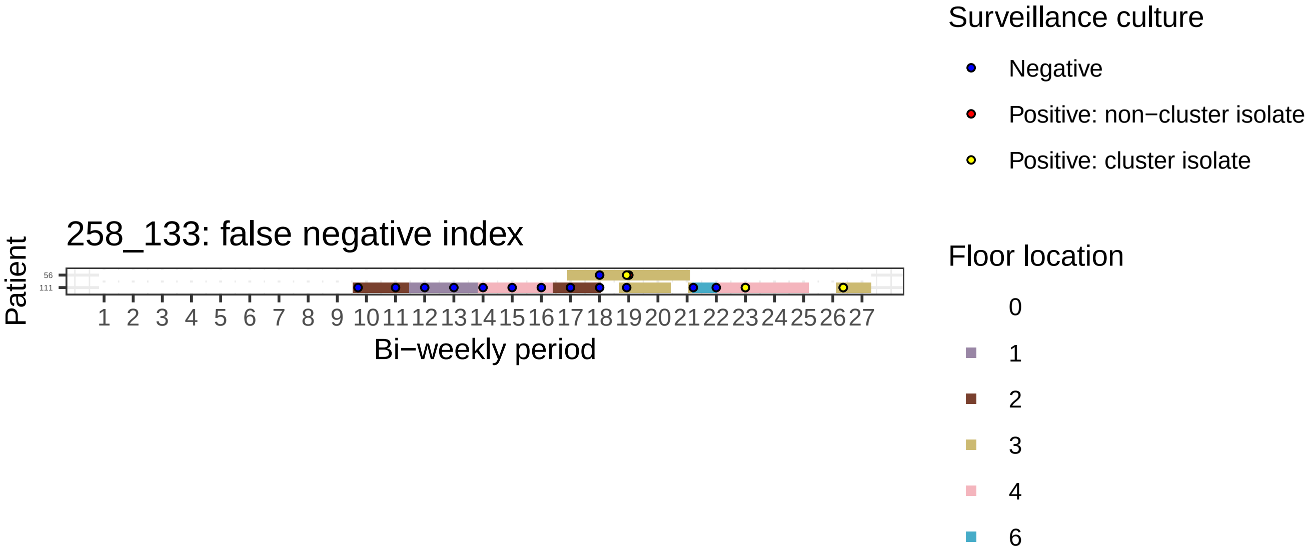

258_133: false negative index
Patient
56
111
1 2 3 4 5 6 7 8 9 10 11 12 13 14 15 16 17 18 19 20 21 22 23 24 25 26 27
Bi−weekly period
Surveillance culture
Negative
Positive: non−cluster isolate
Positive: cluster isolate
Floor location
0
1
2
3
4
6

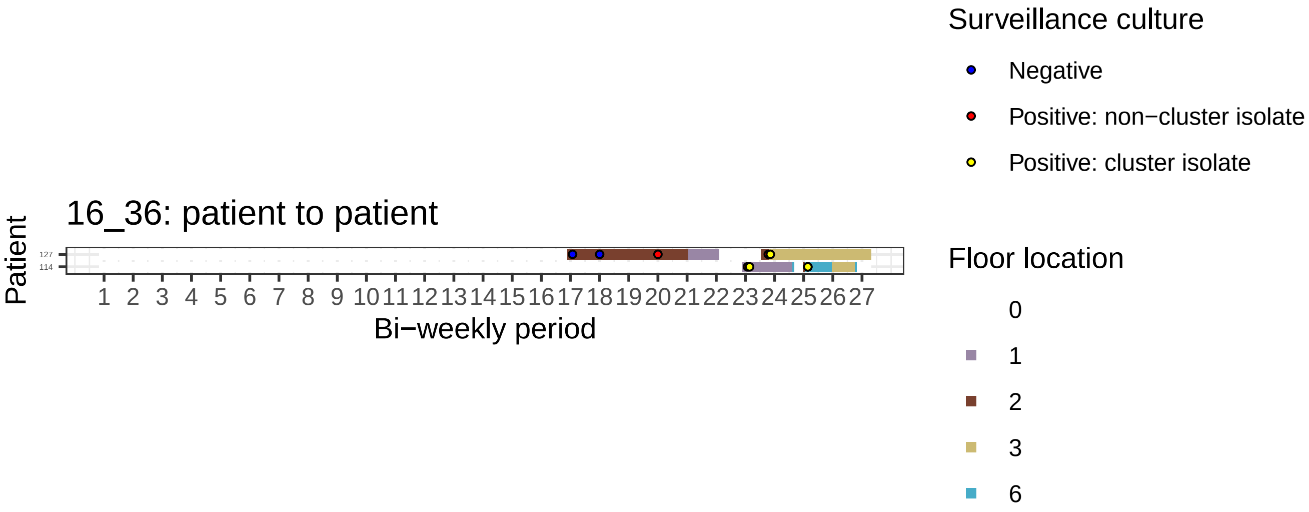
16_36: patient to patient
Patient
127
114
1 2 3 4 5 6 7 8 9 10 11 12 13 14 15 16 17 18 19 20 21 22 23 24 25 26 27
Bi−weekly period
Surveillance culture
Negative
Positive: non−cluster isolate
Positive: cluster isolate
Floor location
0
1
2
3
6

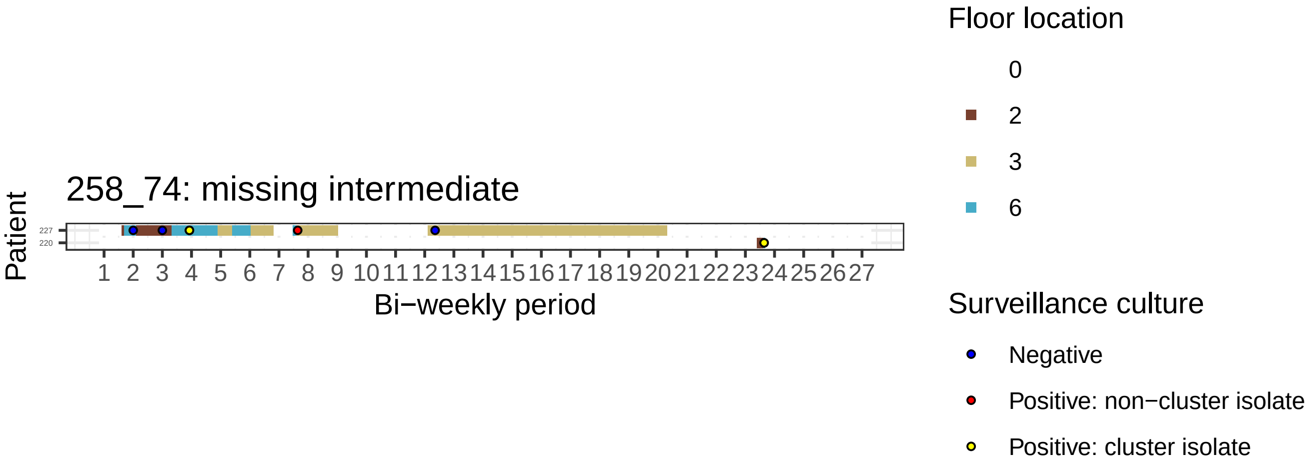

258_74: missing intermediate
Patient
227
220
1 2 3 4 5 6 7 8 9 10 11 12 13 14 15 16 17 18 19 20 21 22 23 24 25 26 27
Bi−weekly period
Floor location
0
2
3
6
Surveillance culture
Negative
Positive: non−cluster isolate
Positive: cluster isolate

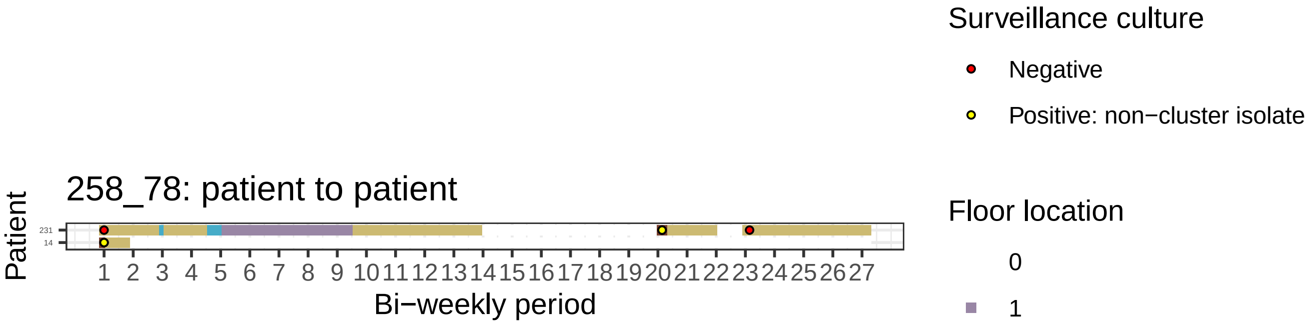

258_78: patient to patient
Patient
231
14
1 2 3 4 5 6 7 8 9 10 11 12 13 14 15 16 17 18 19 20 21 22 23 24 25 26 27
Bi−weekly period
Surveillance culture
Negative
Positive: non−cluster isolate
Floor location
0
1
2
3
6

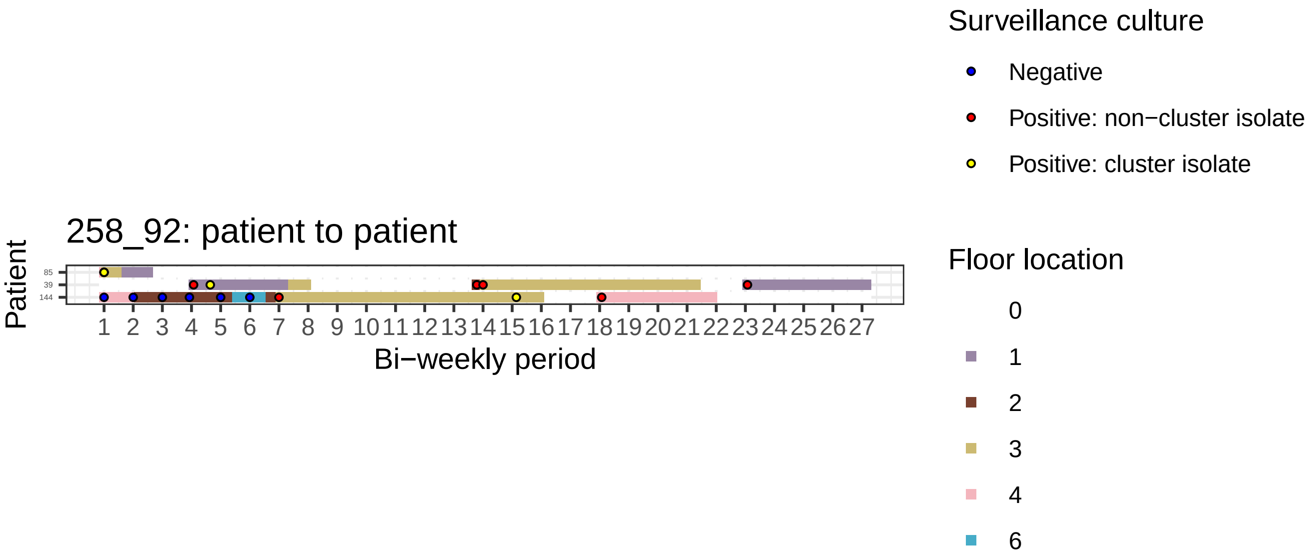

258_92: patient to patient
Patient
85
39
144
1 2 3 4 5 6 7 8 9 10 11 12 13 14 15 16 17 18 19 20 21 22 23 24 25 26 27
Bi−weekly period
Surveillance culture
Negative
Positive: non−cluster isolate
Positive: cluster isolate
Floor location
0
1
2
3
4
6
